# Spatial mapping of polymicrobial communities reveals a precise biogeography associated with human dental caries

**DOI:** 10.1073/pnas.1919099117

**Published:** 2020-05-18

**Authors:** Dongyeop Kim, Juan P. Barraza, Rodrigo A. Arthur, Anderson Hara, Karl Lewis, Yuan Liu, Elizabeth L. Scisci, Evlambia Hajishengallis, Marvin Whiteley, Hyun Koo

**Affiliations:** ^a^Biofilm Research Laboratory, Department of Orthodontics, School of Dental Medicine, University of Pennsylvania, Philadelphia, PA 19104;; ^b^Department of Preventive Dentistry, School of Dentistry, Jeonbuk National University, 54896 Jeonju, Republic of Korea;; ^c^School of Biological Sciences, Georgia Institute of Technology, Atlanta, GA 30332;; ^d^Emory-Children’s Cystic Fibrosis Center, Atlanta, GA 30322;; ^e^Center for Microbial Dynamics and Infection, Georgia Institute of Technology, Atlanta, GA 30332;; ^f^Preventive and Community Dentistry Department, Dental School, Federal University of Rio Grande do Sul, Porto Alegre, RS 90035-003, Brazil;; ^g^Department of Cariology, Operative Dentistry and Dental Public Health, Oral Health Research Institute, Indiana University School of Dentistry, Indianapolis, IN 46202;; ^h^Department of Anatomy and Cell Biology, Indiana University School of Medicine, Indianapolis, IN 46202;; ^i^Divisions of Pediatric Dentistry and Community Oral Health, School of Dental Medicine, University of Pennsylvania, Philadelphia, PA 19104;; ^j^Center for Innovation and Precision Dentistry, School of Dental Medicine and School of Engineering and Applied Sciences, University of Pennsylvania, Philadelphia, PA 19104

**Keywords:** dental caries, polymicrobial, biogeography, biofilm, *Streptococcus mutans*

## Abstract

Tooth decay (dental caries) is a widespread human disease caused by microbial biofilms. *Streptococcus mutans*, a biofilm-former, has been consistently associated with severe childhood caries; however, how this bacterium is spatially organized with other microorganisms in the oral cavity to promote disease remains unknown. Using intact biofilms formed on teeth of toddlers affected by caries, we discovered a unique 3D rotund-shaped architecture composed of multiple species precisely arranged in a corona-like structure with an inner core of *S. mutans* encompassed by outer layers of other bacteria. This architecture creates localized regions of acidic pH and acute enamel demineralization (caries) in a mixed-species biofilm model on human teeth, suggesting this highly ordered community as the causative agent. Notably, the construction of this architecture was found to be an active process initiated by production of an extracellular scaffold by *S. mutans* that assembles the corona cell arrangement, encapsulating the pathogen core. In addition, this spatial patterning creates a protective barrier against antimicrobials while increasing bacterial acid fitness associated with the disease-causing state. Our data reveal a precise biogeography in a polymicrobial community associated with human caries that can modulate the pathogen positioning and virulence potential in situ, indicating that micron-scale spatial structure of the microbiome may mediate the function and outcome of host–pathogen interactions.

## Significance

Dental caries remains an unresolved public health problem. The etiology is poorly understood, as the oral cavity harbors diverse communities of microorganisms. Using multiple imaging modalities on human teeth from patients with caries, we discovered a microbial community precisely arranged in a corona-like architecture. Moreover, this organization is mediated by the pathogen *Streptococcus mutans* through production of an extracellular scaffold that directs positioning of other oral microbes. We developed a methodology to quantify the spatial structure of microbial communities at the micron scale and found a precise spatial patterning of bacteria associated with localized caries onset. These findings are relevant as we approach the post-microbiome era, whereby quantifying the community structural organization may be essential for understanding microbiome function.

Dental caries is one of the most common human infectious diseases, affecting 2.3 billion people globally, with high prevalence (60 to 90%) among underprivileged children (1, 2). This disease is biofilm- and diet-dependent and characterized by acid damage of the enamel, leading to localized demineralization and eventually cavitation and tooth destruction (3). The primary etiologic agent in caries has long been considered to be the bacterium *Streptococcus mutans*, an avid biofilm-former and acid-producer. *S. mutans* has been clinically associated with the disease from microbiome data (4–7), particularly in severe childhood caries, and has been shown to cause caries in animal models of infection (8). However, highly diverse oral microbial communities are present on tooth surfaces (3, 9–11), and these communities display micron-scale spatial organization (11–15). Yet little is known about how *S. mutans* is spatially organized with other microorganisms within intact biofilms associated with human caries to inflict damage to the host enamel tissue.

Considering that caries occur in a highly localized manner on teeth (16), we hypothesized that *S. mutans* may be arranged nonrandomly with other bacteria in a precise spatial organization to modulate virulence in situ. To investigate this, we developed a multistep imaging and computational pipeline to analyze the spatial structure of intact oral biofilms in diseased patients and determine how the community architecture modulates virulence in an experimental biofilm model. Previous sequencing-based studies showed high abundances of *Streptococcus* and *S. mutans* in the biofilm from severe childhood caries (6, 7); thus, we used taxa-specific probes, including *S. mutans*-specific, *Streptococcus*, and all bacteria fluorescent probes, and confocal imaging to map the spatial arrangement of the microbial communities across multiple length scales (from submillimeter to submicron levels).

Using intact dental biofilms formed on the teeth of toddlers affected by caries, we discovered a unique 3D rotund-shaped architecture composed of multiple species arranged in a corona-like structure with an inner core of *S. mutans* surrounded by outer layers of non-mutans streptococci. To assess the impact of this spatially structured community, we simultaneously analyzed the biofilm architecture, pH microenvironments, and enamel surface properties using a mixed-species biofilm model on human teeth. We found that the rotund architecture created a highly acidic pH region that precisely matched the acute demineralization of the enamel surface, indicating that this highly ordered community is associated with localized caries onset. Mechanistically, construction of this architecture is an active process initiated by the production of an extracellular scaffold by *S. mutans* that directs the positioning of other bacteria by creating physical boundaries to assemble the virulent community arrangement. This structural organization also enhances antimicrobial tolerance and increases bacterial fitness associated with the disease-causing state. Our findings reveal the spatial arrangement of polymicrobial biofilm communities in their native state on diseased human teeth, whereby the biogeography may dictate the positioning of pathogens and the virulence potential in situ associated with severe childhood caries.

## Results and Discussion

### Polymicrobial Community Organization of Biofilm on Teeth in the Disease State.

**Fig. 1.**
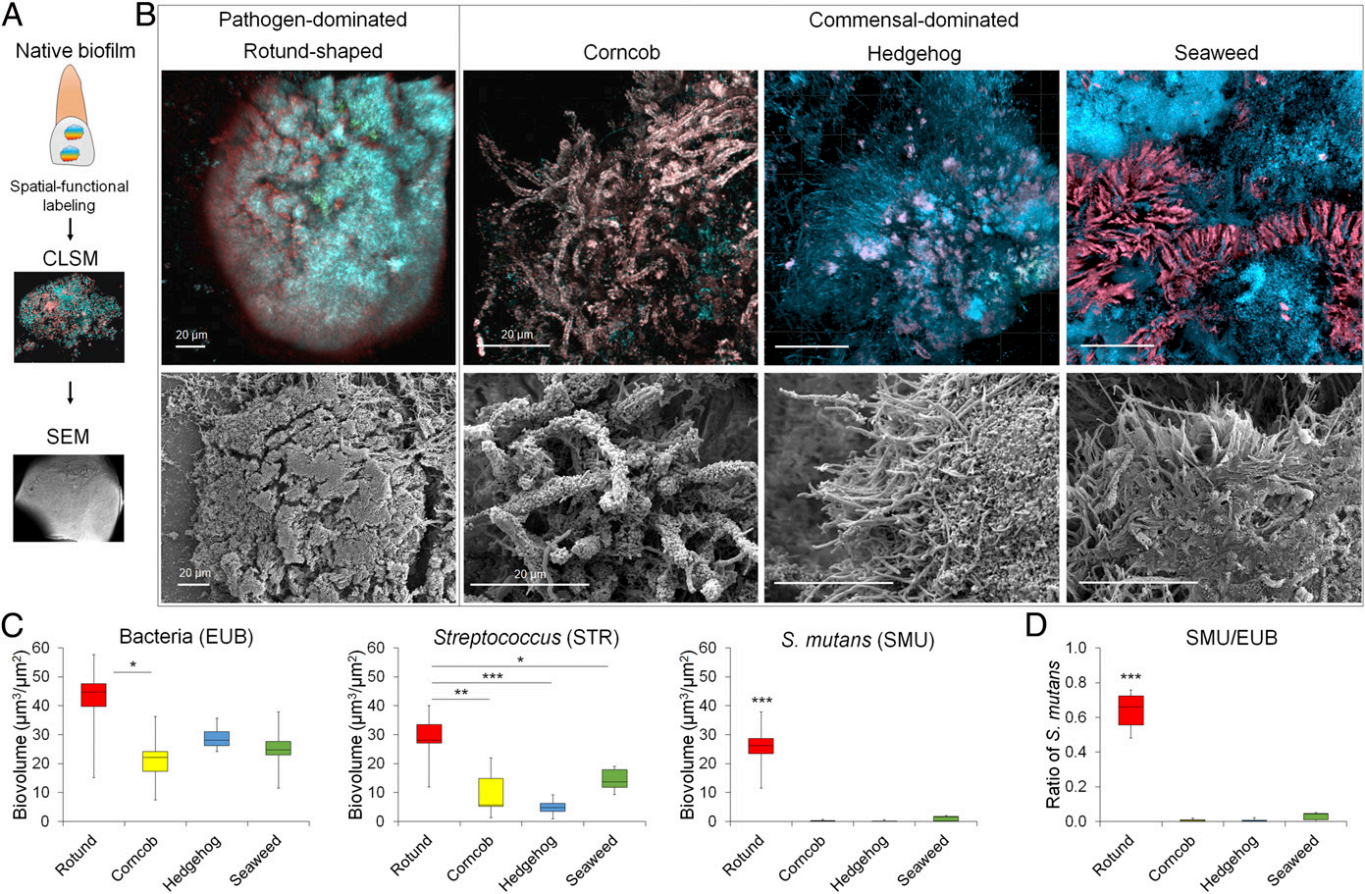
Polymicrobial community organization and architecture of intact plaque biofilm on teeth. (*A*) Intact biofilm structure on teeth followed by fluorescence labeling and confocal imaging and then scanning electron microscopy. (*B*) Distinct spatial organization and morphological patterns (architectures) of ECC plaque biofilms including rotund, corncob, hedgehog, and seaweed-like structures. Rotund was the most commonly detected architecture in the diseased tooth surfaces (found in 21 teeth among 30 samples; 21/30) followed by hedgehog (12/30), corncob (11/30), and seaweed (11/30). Representative images and quantitative imaging analysis of each of the biofilm architectures (in a field of view of 312 × 312 µm^2^ with similar biovolumes of bacteria) were obtained from the following sample sizes: rotund, *n* = 12; corncob, *n* = 10; hedgehog, *n* = 8; and seaweed, *n* = 10. (*C*) Computational quantitative analysis of the microbial composition in each of the biofilm architectures: rotund, *n* = 12; corncob, *n* = 10; hedgehog, *n* = 8; seaweed, *n* = 10. (*D*) Relative abundance of *S. mutans*. In the box-and-whisker plots, the center line denotes the median, whiskers represent minimum and maximum, and box limits denote the 25th and 75th percentiles. *P < 0.05; **P < 0.01; ***P < 0.001, two-tailed Mann–Whitney *U* test.

To analyze the spatial structuring of intact human oral biofilms, it was critical to remove infected teeth from diseased patients without perturbing the biofilm to keep its native state intact (*Materials and Methods*). Once these teeth were collected, we analyzed the spatial and structural organization of naturally formed biofilms on the noncavitated tooth surfaces using confocal laser scanning microscopy and computational analyses after fluorescent labeling of bacteria via taxonomic ranks (domain-genus-species) (Fig. 1*A* and *SI Appendix*, Fig. S1). We then used a fluorescence subtraction method (*SI Appendix*, Fig. S1) to analyze different bacterial arrangements based on taxa-specific labeling. In brief, we used a sequential imaging approach composed of 1) visualization of the entire tooth surface at low magnification using both confocal and scanning electron microscopy; 2) selection of imaging areas to evaluate the biofilm community structure (termed “architecture”) based on morphology and previously used terminologies (11); 3) high magnification, high-resolution imaging of each biofilm architecture and its spatial organization at micron scale; and 4) quantitative computational analysis to determine the biovolume and microbial positioning within the biofilm. A detailed step-by-step protocol of this imaging procedure and a tutorial guide are provided in *SI Appendix*, *Methods*.

Our analysis revealed four types of architecture on the diseased teeth, termed rotund-, corncob-, hedgehog-, and seaweed-like architectures based on their visual morphology and the available terminologies (11, 17) (Fig. 1*B*). These specific terms give an identity to each type of microbial community structure. Rotund was the most commonly detected architecture (found in 21 teeth among 30 samples; 21/30) followed by hedgehog (12/30), corncob (11/30), and seaweed (11/30). In addition, we also performed quantitative imaging analysis (COMSTAT) of the micrographs to determine the microbial content based on taxonomic ranks: all bacteria (EUB), *Streptococcus* (STR), and *S. mutans* (SMU). Corncob and hedgehog were observed previously in plaque samples collected from healthy individuals (11). We also found a seaweed architecture that, similar to corncob and hedgehog, was dominated by nonstreptococcal bacteria and non-mutans streptococci (Fig. 1 *B* and *C*). However, the rotund shape was particularly distinctive. Compared with other types, it exhibited a 3D dome-like structure (Fig. 1*A* and *SI Appendix*, Fig. S2) and relatively high proportions of *Streptococcus* and *S. mutans* (Fig. 1*C*), typically found in sequence-based analyses of plaque from early childhood caries (ECC) (4–7). Interestingly, the rotund shape harbored a greater abundance of *S. mutans* compared with other architectures (∼68% of the total biomass vs. <6%; *P* < 0.001) (Fig. 1*D*). These findings complement a previous study showing the composition and spatial distribution of undisturbed biofilms on natural occlusal caries in which *S. mutans* clusters were found on cavitated lesion sites but not on noncavitated enamel surfaces (18).

### Spatial Organization of the Community across the Rotund Architecture.

**Fig. 2.**
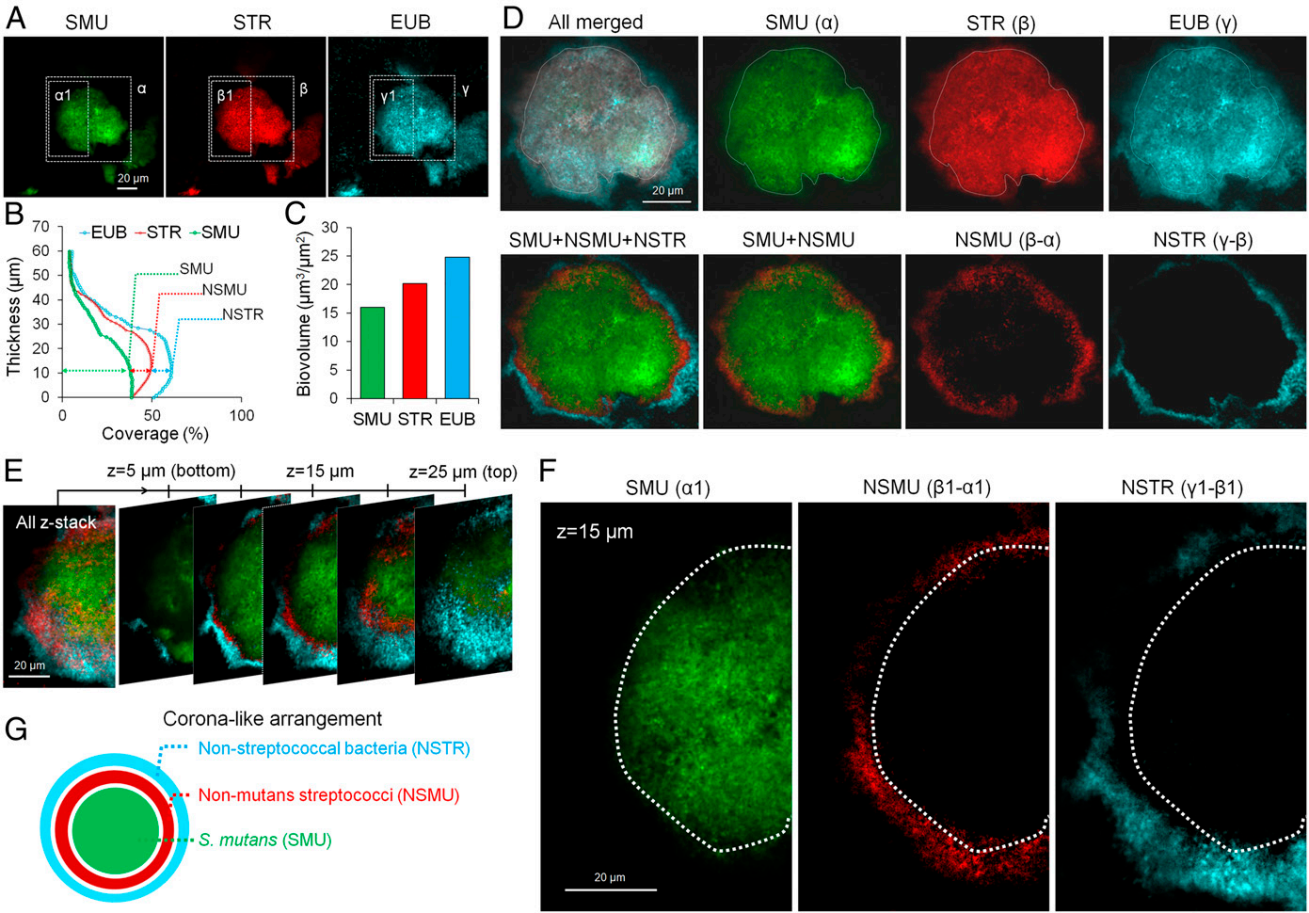
Spatial structuring of the bacterial community across the rotund architecture in its native state. (*A*) Three-channel imaging (species-genus-domain) of the 3D rotund architecture. SMU, *S. mutans*; (green); STR, *Streptococcus* (red); EUB, all bacteria (blue). (*B*) COMSTAT analysis of bacterial cells across the biofilm thickness. (*C*) Quantitative analysis of confocal images for determination of the biomass of SMU, STR, and EUB. (*D*) Computational analysis using a fluorescence subtraction method (details in *SI Appendix*, *Methods*) from taxonomic order as domain-genus-species via ImageJ. (*E*) Z-stack rendering over a 25-μm-thick rotund-shaped microcolony. (*F*) Representative confocal images of a corona-like spatial arrangement of intact biofilms (*z* = 15 μm from the tooth surface) following taxonomic differentiation. Green, SMU; red, NSMU; blue, NSTR. (*G*) Diagram of a corona-like cell arrangement characterized by computational analysis and 3D image rendering.

Given the distinctive 3D architecture, high population of *S. mutans*, and frequency of detection (70% of samples; 21 of 30 teeth), we further examined the spatial structure of the rotund shape (Fig. 2 and *SI Appendix*, Fig. S1*B*). Visualization and quantitative image analyses of the rotund architecture revealed a corona-like assembly composed of a densely packed bacterial cluster (inner core) dominated by *S. mutans* (SMU) that was spatially segregated from outer layers of other oral streptococci (NSMU) and bacteria (NSTR) (Fig. 2). Previous laboratory studies have shown that extracellular polymeric substances (EPS) produced by bacteria can provide structural integrity and scaffolding of 3D biofilm structures (19–21); thus, we next tested for the presence and spatial positioning of bacterial EPS in the rotund architectures. *S. mutans* is an avid producer of an EPS termed glucan synthesized by glucosyltransferases (Gtfs) using sucrose (a key dietary factor in severe childhood caries) as a substrate (3). Using a glucan-specific labeling technique based on Gtf enzymatic activity (21), we were able to assess the spatial production of EPS by the Gtfs present in the human biofilm samples (on extracted teeth) at the time of collection without labeling bacterial cells or its metabolic activity (*Materials and Methods*). We detected functional Gtfs across the structured rotund community, showing glucans generated in close association with bacterial cells (*SI Appendix*, Fig. S2). In contrast, EPS glucans were absent in other architectures. Since segregated lineages usually represent cooperative behaviors of expanding population as clonal clusters (14, 22, 23), corona-like cell segregation of *S. mutans* in the core is likely associated with expansion of a pathogenic cluster embedded in the EPS matrix (21), which could provide stability for the rotund-shaped architecture.

### Rotund Architecture Creates Localized Acidic pH and Enamel Demineralization.

**Fig. 3.**
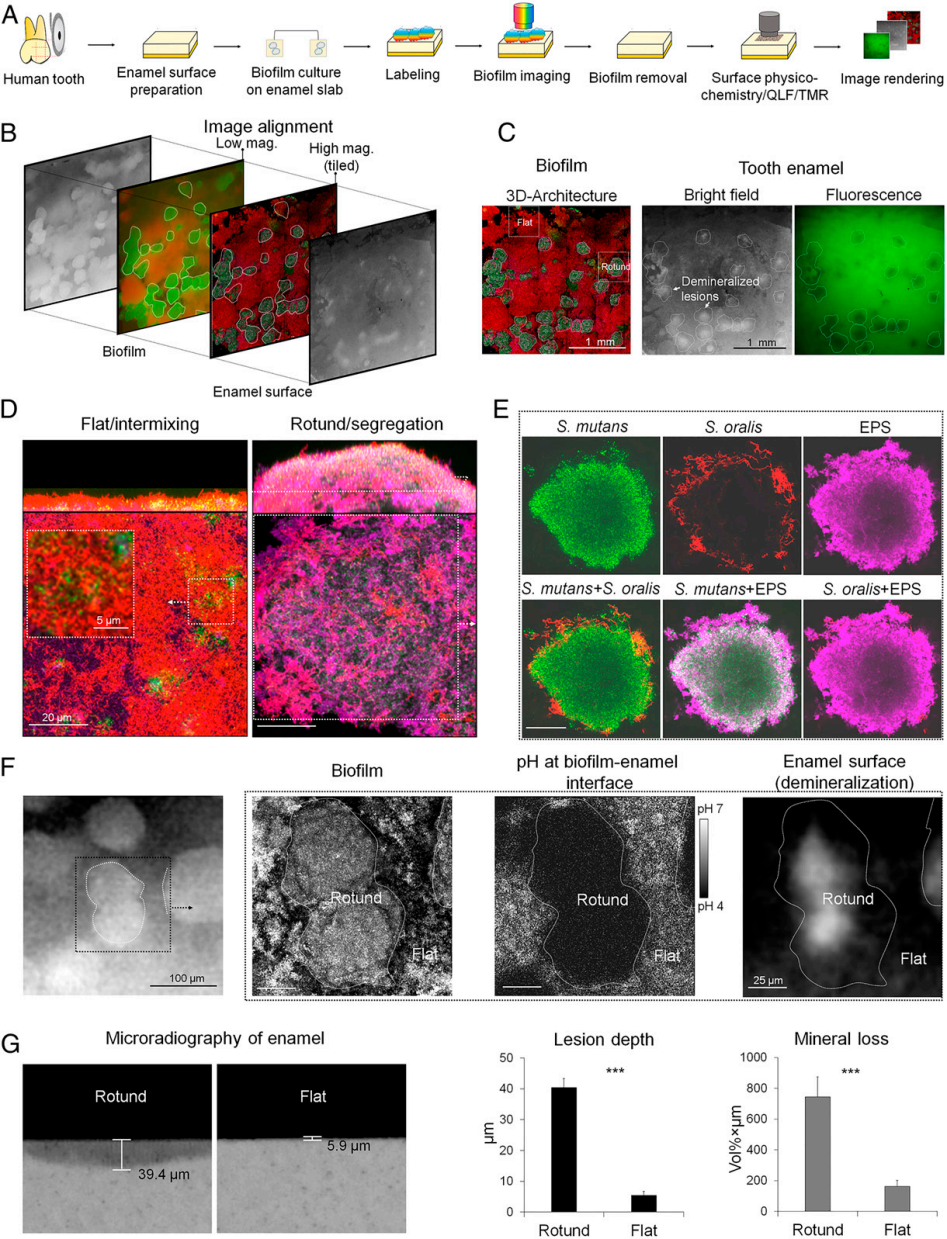
Simultaneous analysis of 3D architecture, pH mapping, and surface physicochemistry. (*A*) Experimental design and workflow of a synchronized analysis for mapping of biofilm architecture, pH distribution, and enamel surface demineralization (optical/fluorescence), followed by lesion depth/mineral loss via TMR (*n* = 8 experiments). (*B*) Simultaneous alignment of biofilm architecture and surface features. (*C*) Confocal image of biofilm 3D architecture on the enamel surface and brightfield and fluorescence images of the surface underneath after biofilm removal. Boundaries (marked with white lines) of rotund-shaped areas are delineated in the confocal image and matched across the enamel surface regions. Demineralized lesions on the enamel surface appear as white spots in the brightfield image and as dark spots in the fluorescence image. (*D*) An example of orthogonal images of rotund and flat communities. (*E*) Close-up images of rotund architecture showing a corona-like cell arrangement in an *S. mutans-S. oralis* biofilm. (*F*) Close-up images showing the rotund-shaped community matched with an acidic pH microenvironment (*Left* and *Middle* in the dotted-line box), whereas in the flat intermixed community, the pH was close to neutral. The acidic pH in the rotund architecture also matched with the localized demineralization of the enamel surface (*Right*). (*G*) Quantitative TMR analysis of the depth of demineralized lesion (µm) and integrated mineral loss (vol% × µm) (rotund, *n* = 7; flat, *n* = 5). Data are mean ± SD. ***P < 0.001, two-tailed Student’s *t* test.

Next, we asked whether the rotund architecture could be assembled in vitro, which would allow for functional studies with the goal of determining the importance of the spatial structure in cariogenesis. As acidification on the tooth enamel surface is a hallmark of caries onset, we were particularly interested in whether the rotund architecture promotes a more acidic environment on the tooth surface (16). The in vitro model we used was a mixed-species biofilm model in which *S. mutans* (pathogen) and *Streptococcus oralis* (commensal) were inoculated on natural human tooth enamel and allowed to form a biofilm in the presence of sucrose (Fig. 3*A*). We developed a sequential stepwise method for synchronized biofilm structure and enamel surface imaging, followed by image realignment for functional assessment related to spatial localization of caries (Fig. 3 *A* and *B*; details provided in *Materials and Methods* and *SI Appendix*, *Methods*). The biofilm structure components were determined via a multilabeling approach using species-specific fluorescent probes and EPS glucan matrix labeling (*Materials and Methods*). In addition, we used our recently developed in situ fluorescence pH mapping method to measure the spatial distribution of pH values across the biofilm structures using ratiometric analysis (24). Finally, we performed enamel surface optical/fluorescence imaging and microradiography to determine acid damage of the enamel surface (i.e., caries lesions). A detailed protocol and a tutorial guide, providing a step-by-step workflow and description of procedures, is provided in *SI Appendix*, *Methods*.

To precisely match the biofilm architectural features with pH mapping and enamel demineralization, we used a hybrid confocal stereomicroscope system using tiled image acquisition to encompass the entire biofilm and enamel surface (Fig. 3 *A* and *B*). This approach allowed synchronized imaging and optical alignment of the biofilm structure formed on the entire enamel block surface with 10-µm-length-scale precision. The human enamel blocks were prepared to have a uniform size (4 × 4 mm^2^) with distinctive edges as fiduciary marks (*SI Appendix*, Fig. S3*A* and *Methods*) to facilitate optical alignment. In brief, the entire biofilm was first imaged using a hybrid confocal-stereomicroscopy system. Then the biofilm was removed from the enamel block and placed back for surface analysis via optical and fluorescence imaging. The enamel placement was guided by the fiduciary marks under the microscope, allowing spatial mapping and alignment between the community structure and demineralized enamel lesions underneath the biofilm.

The 3D-reconstructed and cross-sectional images of the in vitro model revealed two distinct spatial structures similar to those observed on the extracted human teeth:1) the rotund shape with *S. mutans* forming a densely packed inner core surrounded by an outer shell of *S. oralis* cells (Fig. 3 *C*–*E*) and 2) a flatter community dominated by *S. oralis* with intermixed bacterial cells interspersed between rotund architectures (Fig. 3 *C* and *D*). The rotund architecture was also characterized by the presence of an abundant EPS glucan matrix both within and surrounding the segregated pathogen cluster (Fig. 3 *D* and *E*), suggesting that the production of extracellular matrices in situ is likely an important contributor to the biogeography and structural stability of rotund-shaped architectures.

To determine whether the rotund architecture is associated with cariogenic conditions (i.e., high acidity and localized demineralization), we first mapped the location of mixed-species biofilm architectures on enamel surface and the optical (brightfield)/fluorescence image of the surface underneath after biofilm removal (Fig. 3*C*). The demineralized regions in the brightfield (Fig. 3*C*, *Middle*) showed striking similarities to the “white spot” patterns seen in early stages of caries onset in children (16), which appear as localized dark spots (Fig. 3*C*, *Right*) under the fluorescence imaging commonly used in the clinic (25). Overall, we found that the position of the rotund-shaped communities matched the location of caries lesions on the enamel surface (*SI Appendix*, Fig. S4).

We next aligned the biofilm structure with fluorescent pH mapping and enamel surface imaging (Fig. 3*F*). Close-up images revealed that the rotund-shaped community was associated with highly acidic pH microenvironment (Fig. 3*F*, *Left* and *Middle* in the dotted-line box, and *SI Appendix*, Fig. S3*A*), whereas in the flat intermixed community, the pH was close to neutral. The acidic pH in the rotund architecture corresponded to the localized demineralization of the enamel surface (Fig. 3*F*, *Right* and *SI Appendix*, Fig. S3*A*). In addition, we used the same enamel blocks and positioning to analyze phosphate content on the demineralized areas using a confocal Raman microscope. A polarized Raman spectra heatmap based on phosphate intensity confirmed lower amounts of enamel phosphate minerals in the white spot areas compared with nondemineralized regions (*SI Appendix*, Fig. S3*B*).

Following the biofilm-enamel surface analysis, the enamel was retrieved and sectioned transversally for measurement of demineralized lesion depth via quantitative transverse microradiography (TMR). Before sectioning, the region of interest was selected based on the realigned biofilm-surface images encompassing both demineralized and nondemineralized areas (*SI Appendix*, Fig. S3*A* and *SI Methods*, Fig. 2*C*, and ref. 14). Consistent with physicochemical changes associated with the rotund architecture, the enamel lesion depth and mineral loss were also increased (Fig. 3*G* and *SI Appendix*, Fig. S3*C*). Conversely, areas dominated by *S. oralis* in flat, nonrotund structures (despite similar bacteria biomass as rotund structures; *SI Appendix*, Fig. S4) did not cause enamel demineralization (Fig. 3*G* and *SI Appendix*, Fig. S3*C*). The rotund architecture created highly acidic pH regions at the biofilm–enamel interface, resulting in localized surface coarsening and light scattering and leading to delineated areas of eroded enamel with an opaque and white chalky appearance, as typically seen in clinical caries and acid erosion of other mineralized tissues and surfaces (16, 26).

### How Does the Rotund Architecture Form?

**Fig. 4.**
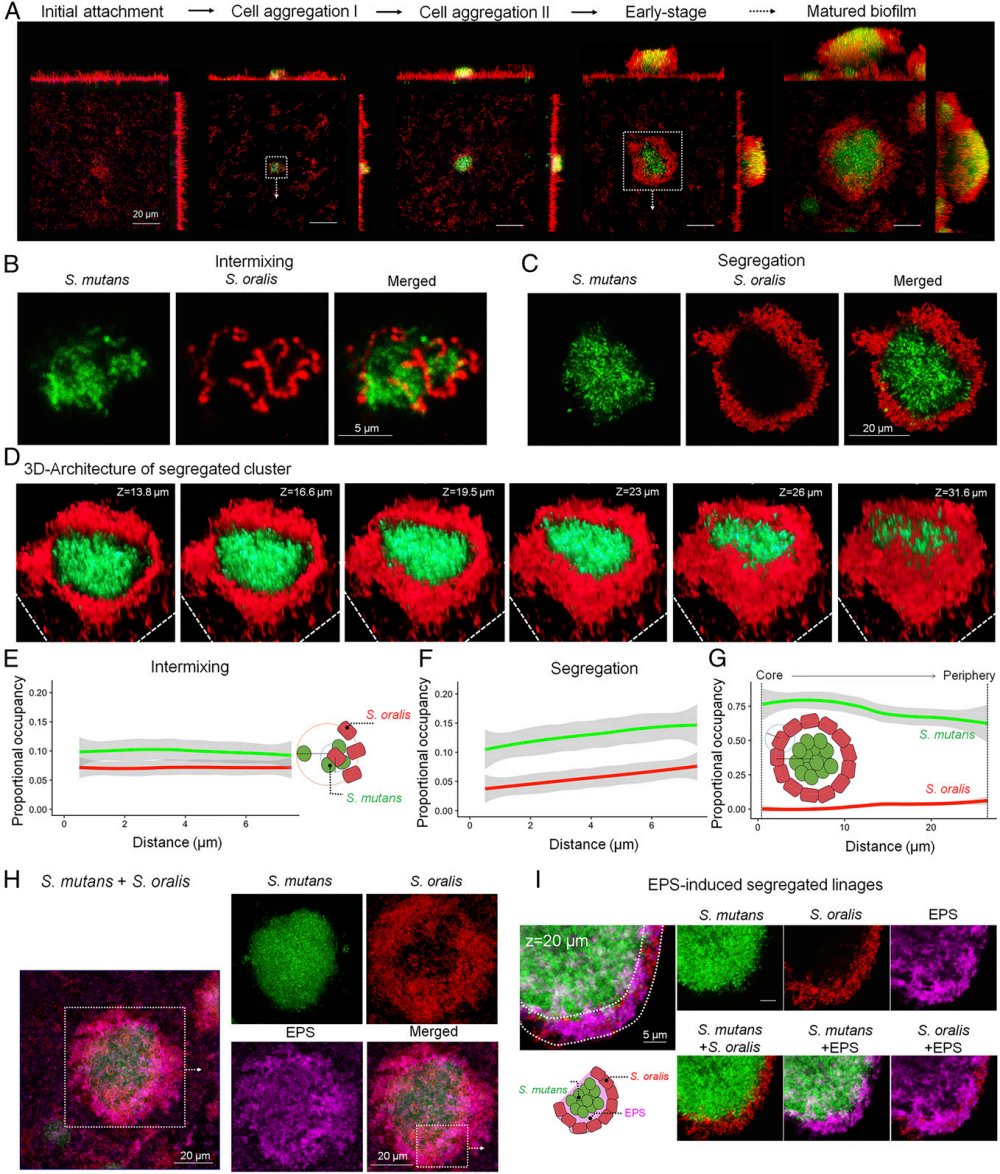
Spatial positioning and 3D structural organization of rotund-shaped architecture. (*A*) Spatiotemporal changes in the patterns of bacterial cell arrangement. Confocal images show the dynamics of spatial organization and cell arrangement between *S. mutans* and *S. oralis* during biofilm development. (*B* and *C*) Confocal images of cell intermixing at early stages of community development (*B*) and the assembly of a spatially segregated corona-like cell arrangement at later stage of the biofilm (*C*). (*D*) 3D reconstruction of the rotund-shape architecture. (*E* and *F*) PO of *S. mutans* using *S. oralis* as a focal point (green) and *S. oralis* using *S. mutans* as a focal point (red) in intermixed (*E*) and segregated (*F*) phases of biofilm development (*n* = 6). (G) Core/periphery calculations performed using a centroid of the *S. mutans* biomass as the focal point and then calculating the PO of *S. mutans* (red) and *S. oralis* (green) (*n* = 6). Shaded areas depict mean ± SD. (*H* and *I*) Spatial cell segregation in rotund-shaped architecture with a corona-like cell arrangement via species-specific and EPS glucan labeling (*H*) and close-up image of EPS-induced segregated linages (*I*).

To further understand the mechanisms of bacterial positioning and cell arrangement in development of the rotund architecture, we examined the spatiotemporal organization patterns using time-lapse imaging and computational analysis. We observed that the community was predominately intermixed with flat architecture at early stages and progressively underwent structural changes toward the rotund-shaped architecture with spatially segregated communities (Fig. 4 *A*–*C*). A 3D analysis of this structure revealed *S. mutans* clustered in the center, forming an inner core, with *S. oralis* surrounding its periphery across the entire depth (Fig. 4*D*) arranged in a “dome-shaped shell” wrapping the pathogen cluster.

To quantitatively characterize the spatial organization of these two distinct communities (intermixed and segregated) at the micrometer scale, we calculated the proportional occupancy (PO) using a customized algorithm (*Materials and Methods*). PO is defined as the proportion of the volume occupied by a target entity (e.g., bacterial cell) at discrete distance intervals away from a focal entity (e.g., another bacterial cell). These discrete volume intervals are analogous to concentric shells of increased radius centered around a focal point and ultimately characterize how the entire population changes around a focal object as one moves away from it. Calculating the PO for multiple bacteria and EPS in a single image quantitatively characterizes the overall spatial organization of the community. Using this metric to analyze the rotund architecture, we found that the PO of *S. oralis* increased along with distance when *S. mutans* was used as the focal point, and that the PO of *S. mutans* increased with distance when *S. oralis* was used as the focal point (Fig. 4 *E* and *F*). This indicates a nonrandom pattern of spatial organization between *S. mutans* and *S. oralis,* demonstrating that *S. oralis* is physically separated from the *S. mutans* core (Fig. 4 *F* and *G*). Conversely, the PO did not change across distance in the flat-intermixed cell arrangement, indicating that *S. mutans* and *S. oralis* were randomly organized and close to each other (intermixing) (Fig. 4*E*). In addition, we found that *S. mutans* was closely associated with the EPS matrix in the flat architectures, since PO between both entities decreased with increasing distance (*SI Appendix*, Fig. S5). However, when the community shifted to the rotund architecture, EPS became more abundant in the community and enmeshed *S. mutans*, while *S. oralis* was positioned at the outer layer of the *S. mutans*-EPS cluster (Fig. 4 *H* and *I*). This suggests that *S. mutans* uses EPS to build a scaffold that subsequently allows for formation of the rotund architecture.

Given the EPS location and structural organization in the mixed community, we assessed the changes in the expression of specific genes associated with biofilm formation over time as the community structure shifts from intermixing to segregation. We focused on key genes involved in glucan production (*gtfB*) and glucan-binding protein (*gbpC*) that are essential for 3D scaffolding and structural integrity through physical associations between bacteria and the EPS matrix (27, 28). We found a significant up-regulation of *S. mutans gtfB* (threefold increase; *P* < 0.001) in the rotund architecture compared with the flat architecture (*SI Appendix*, Fig. S6). We then examined whether a strain defective in *gtfB* can alter the interspecies spatial arrangement and the assembly of rotund architecture (*SI Appendix*, Fig. S7*A*). Since the *gtfB* mutant and wild-type *S. mutans* have similar growth rates (*SI Appendix*, Fig. S7*B*), we were able to determine the role of EPS in constructing the rotund and flat architectures. We found that an *S. oralis* and *gtfB*-defective *S. mutans* community was unable to form the rotund architecture and exhibited a disordered intermixing spatial pattern (*SI Appendix*, Fig. S7 *C* and *D*). Conversely, supplementation with GtfB promoted clustering of *S. mutans* surrounded by outer layers of *S. oralis*, leading to reestablishment of the rotund architecture with a corona-like arrangement (*SI Appendix*, Fig. S7*C*). However, the *S. oralis* corona-like positioning was dependent on the location on the apatitic surface, whereby in some areas the *S. oralis* had started to encompass the *S. mutans* cluster, while in other regions it was fully completed, forming the corona arrangement with the pathogen. Quantitative analysis of the bacterial positioning based on PO confirmed the visual observations, indicating the importance of GtfB in assembling a nonrandom and structured mixed community (*SI Appendix*, Fig. S7*D*).

**Fig. 5.**
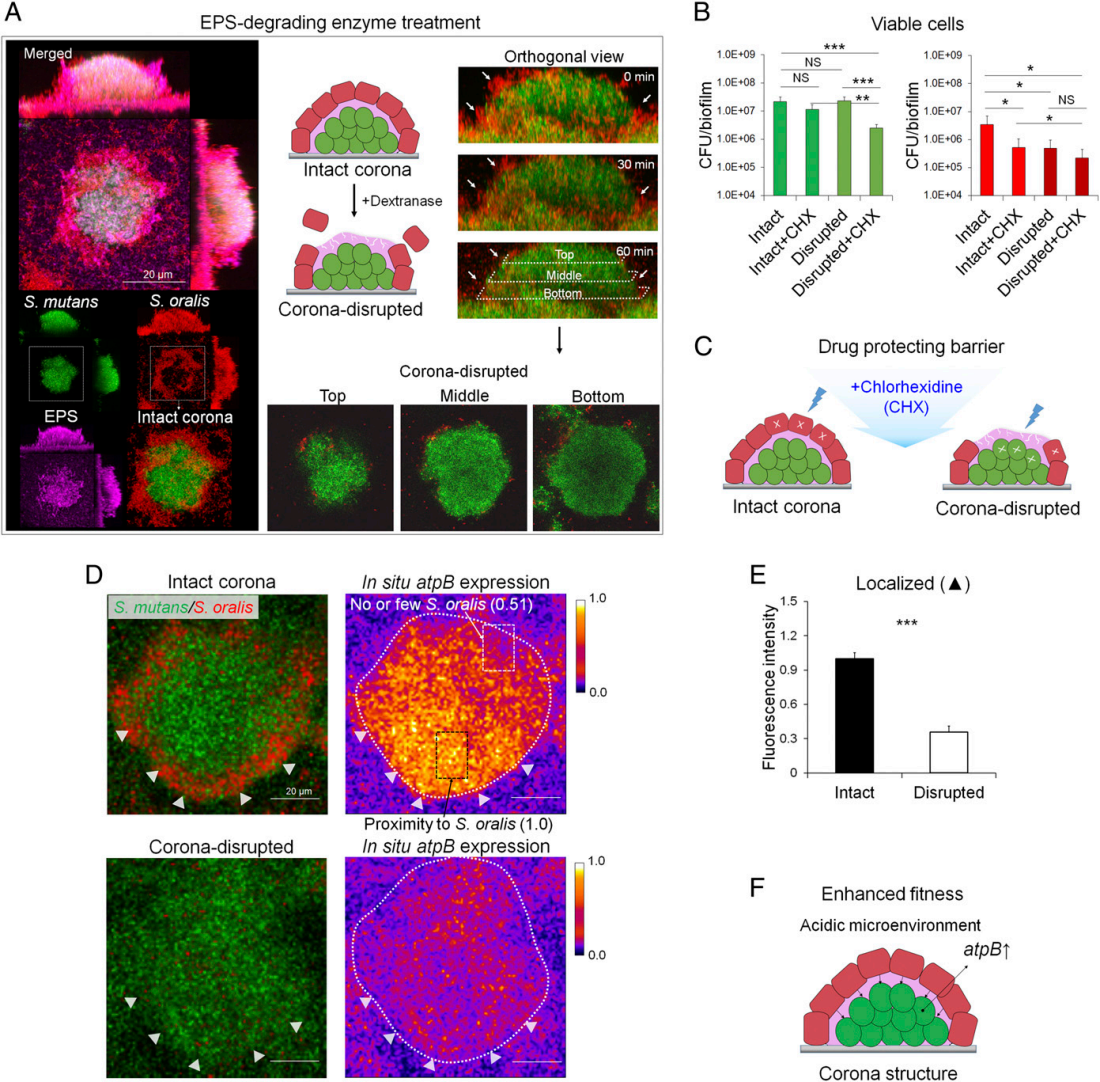
Effect of corona community structure on antimicrobial tolerance and fitness of *S. mutans*. (*A*) Disruption of the corona-like cell arrangement via EPS-degrading enzyme (dextranase) treatment. (*B*) Viable cell counts (CFU) of *S. mutans* and *S. oralis* recovered from the biofilms with either intact (buffer-treated control) or disrupted (dextranase-treated) corona structure following topical treatment with 0.12% CHX or PBS (as vehicle control vs. CHX) (*n* = 6). Data are mean ± SD. NS, not significant. *P < 0.05; **P < 0.01; ***P < 0.001, two-tailed Student’s *t* test. (*C*) Proposed “drug-protecting barrier” for antimicrobial tolerance of *S. mutans* afforded by the intact corona structure. (*D*) In situ *atpB* gene expression of *S. mutans* within an intact corona structure (buffer-treated control) and a disrupted corona structure (dextranase-treated). Arrowheads indicate the periphery of the *S. mutans* microcolony surrounded by *S. oralis* (*Upper*) or disrupted physical association with *S. oralis* (*Lower*). Boxes and arrows on the right (*atpB* expression) indicate site-specific in situ gene expression of *S. mutans* cells located either in proximity to the *S. oralis* outer ring or next to none or few *S. oralis* cells nearby. (*E*) Localized gene expression level (arrowheads indicate locations) via measurement of GFP fluorescence intensity using ImageJ (*n* = 15). Data are mean ± SD. ***P < 0.001, two-tailed Student’s *t* test. (*F*) Enhanced fitness of *S. mutans* with an intact corona structure under the acidic microenvironment as determined by increased *atpB* gene expression.

To further validate this GtfB glucan-mediated mechanism, we added EPS-degrading dextranase to digest glucans or a Gtf inhibitor to inhibit glucan synthesis without affecting bacterial viability (29). Dextranase is devoid of antibacterial activity or effects on metabolic activity, as demonstrated previously (24, 30). Exposure to dextranase resulted in degradation of the EPS matrix (*SI Appendix*, Fig. S8), which disassembled the corona-like layers of *S. oralis* cells without disturbing the structural integrity of the *S. mutans* inner core and its cell viability compared with enzyme-buffer treated control (intact corona; *SI Appendix*, Fig. S8). Close-up images of corona disruption across the biofilm are shown in Fig. 5*A* (*Bottom*). Likewise, topical treatment with povidone iodide 0.25% to 0.5%, concentrations that inhibit EPS synthesis without affecting cell viability compared with vehicle (PBS) control, on a mixed-species biofilm reduced glucan synthesis and disrupted the corona-like arrangement with *S. oralis* (*SI Appendix*, Fig. S9), demonstrating the importance of EPS glucans for establishing this spatial structure.

### Corona Community Structure on Antimicrobial Tolerance and Bacterial Fitness.

Based on the precise biogeography of the rotund architecture with *S. mutans* in the core, we assessed whether this spatial structuring would protect the pathogen against antimicrobials. We examined the antimicrobial susceptibility of biofilms with an intact and a disrupted community structure using chlorohexidine (CHX), a clinically used oral antimicrobial agent. Dextranase was used to disrupt the corona-like cell arrangement (as described above), and the efficacy of CHX was compared with intact structure (control) based on viable cell counts and a time-lapse live/dead assay (30). In brief, biofilms with intact (enzyme buffer-treated) or disrupted (dextranase-treated) corona were prepared and then exposed to 0.12% CHX or vehicle control (PBS) for 5 min. Then viable cell counts (CFU) of *S. mutans* and *S. oralis* for each of the experimental conditions were determined (Fig. 5*B*). For *S. mutans*, intact and corona-disrupted biofilms treated with vehicle (PBS) displayed similar numbers of viable cells. In intact biofilms, CHX treatment was ineffective against *S. mutans* (vs. vehicle) but was capable of reducing the viability of *S. oralis* (*P* < 0.05). However, the corona-disrupted biofilm displayed increased susceptibility of *S. mutans* to CHX treatment (vs. vehicle, *P* < 0.001), resulting in fewer viable cells (vs. intact biofilm treated with CHX, *P* < 0.01) (Fig. 5*B*). These findings were complemented by cell viability imaging (*SI Appendix*, Fig. S10). Thus, the intact corona structure can provide enhanced antimicrobial tolerance of *S. mutans* cells located in the inner core (Fig. 5*C*).

Since the rotund architecture creates a protective environment, it is conceivable that *S. mutans* cells in the inner core may also display enhanced fitness in low-acid pH environments. To address this possibility, we used biofilms with intact (buffer-treated) or disrupted (dextranase-treated) architecture and performed simultaneous pH mapping and in situ *atpB* (a key gene associated with acid tolerance) expression analysis, as described previously (24). Under our experimental conditions, dextranase did not affect in situ pH (compared with intact, buffer-treated control; *SI Appendix*, Fig. S11) or bacterial activity. In intact biofilm, we found that *atpB* expression was induced at pH 4.5, compared with pH 7.0 in disrupted biofilm (*SI Appendix*, Fig. S11), consistent with previous studies (24, 31, 32). Notably, *atpB* expression was elevated in *S. mutans* cells located in proximity to the *S. oralis* outer layer compared with those with no or few *S. oralis* cells nearby across intact architecture (arrows in Fig. 5*D*). However, we found a distinctive gene expression pattern when comparing intact (buffer-treated control) and corona-disrupted (dextranase-treated) architectures. The *atpB* expression by *S. mutans* was significantly higher within intact rotund architecture compared with dextranase-perturbed architecture at both pH 4.5 and pH 7.0 (Fig. 5*D* and *SI Appendix*, Fig. S11). Furthermore, quantitative fluorescence analysis revealed 2.8-fold higher gene expression (Fig. 5*E*) by *S. mutans* within the intact corona structure compared with the disrupted structure (arrowheads in Fig. 5*D*). These data indicate that, in addition to antimicrobial protection, the intact rotund architecture and *S. oralis* corona promotes acid tolerance of *S. mutans*, an important fitness trait for creating a virulent (cariogenic) microenvironment (Fig. 5*F*).

Recently, *S. oralis* and other mitis streptococci have been classified as accessory pathogens (i.e., commensal bacteria that may enhance the virulence of pathogens) in periodontitis (33). However, *S. oralis* and *S. mutans* also have been shown to antagonize each other via bacteriocins and hydrogen peroxide production (34, 35), indicating tightly regulated cooperative and competitive interactions to mediate coexistence and survival within biofilms. Whether its spatial positioning with *S. mutans* influences the virulence in vivo awaits further experimental validation using a rodent caries model.

**Fig. 6.**
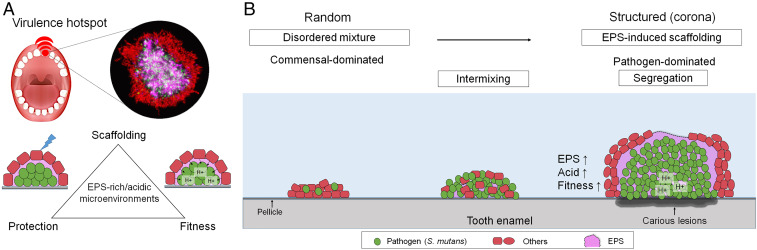
A proposed model of a virulence hotspot associated with precise biogeography. (*A*) Pathogens such as *S. mutans* form highly organized polymicrobial communities in humans, with precise biogeography dictating its positioning at the infection site and creating virulent microenvironments. (*B*) Spatiotemporal structuring and community organization leading to the assembly of an inner bacterial core composed of *S. mutans* clusters surrounded by EPS glucan matrix and outer layers of other oral bacteria, such as *S. oralis*, leading to localized onset of demineralized enamel lesions.

In summary, spatial mapping of the intact polymicrobial biofilm communities revealed a precise biogeography associated with human caries. A unique rotund-shaped architecture composed of multiple species in corona-like arrangement stood out as a landmark communal organization on the infected teeth. Further analysis revealed a dense accumulation of *S. mutans* (a cariogenic pathogen) clustered in the center, forming an inner core encapsulated by outer rings of non-mutans streptococci and other bacteria in a highly ordered manner. Using an experimental biofilm model, we found that this spatial patterning is an active process mediated by the pathogen via production of an extracellular scaffold, which provides a protective milieu whereby *S. mutans* displays enhanced antimicrobial and acid tolerance. In turn, the rotund architecture creates a localized acidic pH microenvironment at the biofilm–tooth interface that causes caries development in situ. In the structured polymicrobial communities, mapping the pathogen positioning and the acidic microenvironments in their native diseased state may reveal unique “virulence hotspots” associated with severe childhood oral disease (Fig. 6). Our findings highlight the importance of biogeography of the human microbiome, whereby the spatial structuring of pathogens and commensal microbiota may dictate the virulence potential in situ. This conceptual framework may be applicable to other biofilm-associated polymicrobial diseases in humans and may provide important insights to aid the development of therapies targeting this precise biogeography.

## Materials and Methods

### Sample Collection and Ethics Statement.

To assess the biofilm architecture in disease, we examined the spatial organization of the microbial community using extracted teeth from patients affected by dental caries. Tooth samples (30 teeth) were extracted from children (age 36 to 72 mo) diagnosed with severe ECC as defined by the 2014 Conference Manual of the American Academy of Pediatric Dentistry. Tooth extraction is required if the child has tooth decay extending to the pulp and periapical infection, and if functionality cannot be restored. Primary maxillary anterior teeth with mesial or distal carious lesions were extracted using a pediatric maxillary anterior forceps adapted to grasp mesiodistally so as to not disturb the dental plaque formed on the buccal surface of the tooth enamel. The teeth were luxated and extracted by applying mesiodistal rotational forces, and the intact biofilm was immediately transferred to a custom plate to immobilize the teeth and preserve the integrity of the biofilm structure. Tooth samples that could not be extracted using the foregoing method were not included for further analyses. A highly experienced pediatric dentist who was blinded to the study performed the tooth extractions. For imaging purposes, we excluded the sample if >30% of the enamel surface was cavitated or missing and if the clinician was unable to extract the tooth while preserving the intact biofilm. Whole saliva was collected for the purpose of coating the hydroxyapatite or tooth enamel blocks for the in vitro biofilm studies.

Ethical approval and written consent/permission forms were obtained from the University of Pennsylvania’s Institutional Review Board (IRB #824243) before the start of the study. For each child, a permission form was reviewed and signed by a legal guardian. All subjects provided written informed consent (no children participated in the saliva collection) under the protocol reviewed and approved by the University of Pennsylvania’s Research Subject Committee (IRB #818549).

### Spatial Organization of Microbial Communities in Intact Biofilms on the Tooth Surface.

The intact and undisturbed biofilms formed on noncavitated tooth surfaces were gently washed twice with PBS and fixed with 4% paraformaldehyde (in PBS, pH 7.4) at 4 °C for 4 h. After fixation, the biofilms were washed twice with PBS, then transferred into 50% ethanol in PBS (pH 7.4), and stored at −20 °C. The biofilm 3D architecture was analyzed via fluorescence in situ hybridization (FISH) as detailed previously (36, 37). The following FISH oligonucleotide probes were used in this study: MUT590, 5′-ACT​CCA​GAC​TTT​CCT​GAC-3′ with Alexa Fluor 488 for *S. mutans*; STR405, 5′-TAG​CCG​TCC​CTT​TCT​GGT-3′ with Cy5 for *Streptococcus*; and EUB338, 5′-GCT​GCC​TCC​CGT​AGG​AGT-3′ with Cy3 for all bacteria. The sample in the hybridization buffer (25% formamide, 0.9 M NaCl, 0.01% SDS, and 20 mM Tris⋅HCl pH 7.2) with the probes was incubated at 46 °C for 4 h. After incubation, the hybridized cells were washed with washing buffer (0.2 M NaCl, 20 mM Tris⋅HCl pH 7.5, 5 mM EDTA, and 0.01% SDS) and incubated for another 15 min at 46 °C. A detailed step-by-step protocol and procedure are provided in *SI Appendix*, *Methods*.

### Bacterial-Derived EPS Analysis of Intact Biofilms.

Major EPS components in cariogenic biofilms are extracellular glucans produced by Gtfs that are secreted extracellularly and bound to microbial surfaces (3). The localized Gtf activity was determined by incorporation of a dextran (10 kDa)-conjugated fluorescent indicator into glucans in the presence of a sucrose substrate (38). The fluorescently labeled dextran acts as a primer and is incorporated into newly formed glucan without staining bacterial cells (38). Freshly extracted teeth were gently immersed in 2 mL of a sucrose substrate (200 mM sucrose, 40 µM dextran 900, and 0.02% NaN_3_ in a buffer consisting of 50 mM KCl, 1 mM CaCl_2_, and 0.1 mM MgCl_2_, pH 6.5) containing a 1 μM Alexa Fluor 647-dextran conjugate (Molecular Probes). The reaction mixture was incubated at 37 °C for 18 h. After incubation, the fluorescently labeled Gtf-EPS glucans were observed using confocal microscopy. The presence of NaN_3_ in the reaction mixture inhibits bacterial metabolic activity as determined experimentally, allowing visualization and measurement of EPS glucans derived from extracellular activity of Gtf in situ. Since bacterial metabolism is inhibited by the addition of sodium azide, we determined EPS synthesized by the Gtfs present in the sample at the time of collection rather than by de novo secretion from bacteria growing during the incubation period with the sucrose substrate. An EPS glucan-labeled sample was then prepared for FISH procedures for microbial cell labeling as described above.

### In Vitro Biofilm Model.

Biofilms were formed on saliva-coated hydroxyapatite (sHA) discs or human enamel blocks vertically suspended in 24- or 48-well plates using a custom-made wire specimen holder, mimicking the smooth surfaces of the pellicle-coated tooth (21, 38). For an in vitro model, well-characterized cariogenic streptococci (*S. mutans* UA159; ATCC 700610) and early colonizing commensal streptococci (*S. oralis*; ATCC 35037) were grown in ultrafiltered tryptone-yeast extract broth (UFTYE; 2.5% tryptone and 1.5% yeast extract, pH 7.0) with 1% glucose at 37 °C and 5% CO_2_ to exponential phase. Each bacterial suspension was then mixed to provide an inoculum with a defined microbial population of *S. mutans* (10^5^ CFU mL^−1^) or *gtfB-*defective strain (with or without GtfB supplementation) and *S. oralis* (10^7^ CFU mL^−1^). The mixed population was inoculated in 2.8 mL of UFTYE containing 0.1% (wt/vol) sucrose and then incubated for 19 h to form an initial biofilm community on the apatitic surface. Then the biofilms were transferred to UFTYE containing 1% sucrose to induce environmental changes to simulate a cariogenic challenge at 19 h following the ecological plaque biofilm model (21, 39). The culture medium was changed twice daily until the end of the experimental period. Bacterial cells were labeled with the following species-specific FISH probes: MUT590, 5′-ACT​CCA​GAC​TTT​CCT​GAC-3′ with Alexa Fluor 488 or Cy5 for *S. mutans* and MIT588, 5′-ACA​GCC​TTT​AAC​TTC​AGA​CTT​ATC​TAA-3′ with Cy3 for *S. oralis.* The EPS were labeled with 1 μM Alexa Fluor 647 (or Alexa Fluor 488)-dextran conjugate as described previously (36, 38). For corona-disruption experiment, bacteria and EPS-labeled sample was treated with 100 units of dextranase (hydrolyzing α-1,6-glucan, EC 3.2.1.11; Sigma-Aldrich) and performed 4D (*x*, *y*, *z*, *t*) real-time confocal imaging as described previously (30). We also investigated the effect of a Gtf inhibitor, povidone iodide, on the establishment of 3D biofilm architecture. Povidone iodide is a potent inhibitor that can selectively inhibit glucan synthesis by GtfB without affecting bacterial cell viability when used at low concentrations (29). The inhibitor (at 0.25 or 0.5% vol/vol in the PBS vehicle, pH 7.2) was topically applied to the biofilms with a brief 10-min exposure at five times during biofilm development (6, 19, 29, 43, and 53 h); this treatment regimen allowed optimal EPS inhibition without affecting cell viability. After each treatment, sHA discs were dip-washed in sterile PBS solution to remove excess agent and then transferred to fresh culture medium until the end of the experimental period (67 h).

### Biofilm Imaging Using Confocal Laser Scanning Microscopy.

The 3D biofilm architecture was acquired using a confocal laser scanning microscope (LSM 800; Carl Zeiss) equipped with an Airyscan super resolution detector and a 20× (1.0 NA) water immersion objective. The biofilms were sequentially scanned using diode lasers (488, 561, and 640 nm), and the fluorescence emitted was collected with a GaAsP or multi-alkali PMT detector (490 to 550 nm for Alexa Fluor 488, 565 to 620 nm for Cy3, and 645 to 700 nm for Cy5 or Alexa Fluor 647). If the biofilm was >200 μm thick, a multiphoton confocal scanning laser microscope (SP8; Leica Microsystems) equipped with a 20× (1.0 NA) water immersion lens was used. The fluorescently labeled biofilm was excited at 780 nm, and the fluorescence emitted was collected with a non-descanned hybrid (HyD-RLD) (RLD1 for Alexa Fluor 488, 500 to 550 nm; RLD3 for Cy5 or Alexa Fluor 647, 665 to 705 nm; RLD4 for Cy3, 604 to 644 nm) or PMT detector. For planktonic single cells or in homogenized biofilm (for probe verification), images were acquired using an LSM 800 confocal laser scanning microscope equipped with 40× (1.2 NA) oil immersion objective in a sequential scanning as described above or an inverted confocal laser scanning microscope (SP5-II; Leica Microsystems) equipped with a 63× (1.4 NA) oil immersion lens in a sequential scanning using a 488-nm argon laser and 543-nm and 633-nm He-Ne lasers. Amira 5.4.1 software (Visage Imaging) and Zen software (Carl Zeiss) were used to create 3D renderings to visualize the biofilm architecture. A detailed step-by-step protocol and procedures are provided in *SI Appendix*, *Methods*.

### Scanning Electron Microscopy.

Samples were fixed in 2% paraformaldehyde/2% glutaraldehyde overnight and, after rinsing, were gradually dehydrated using increasing concentrations of ethanol (50, 70, 80, 90, and 100% for 10 min each). Samples were then chemically dried with hexamethyldisilazane and sputter-coated (Au/Pd) before imaging. The correlated image with confocal images were acquired using a high-resolution scanning electron microscope (Quanta 250 FEG; FEI).

### Computational Quantitative Image Analysis.

The confocal images were analyzed using COMSTAT for quantification of microbial cells and EPS within intact biofilms (29). The biovolume represents the overall biomass occupied by bacterial cells (bacteria-*Streptococcus*-*S. mutans*) or EPS within intact biofilms, which provides a direct measurement of their amounts and ratios across the biofilm depth (from tooth surface to fluid phase). For spatial cell arrangement (positioning of *S. mutans* across the microbial consortium) within the polymicrobial biofilm structure, channel subtraction was applied through classification of a set of elements using the ImageJ Image Calculator: SMU, *S. mutans* alone; STR – SMU = non-mutans streptococci (NSMU); EUB – STR = nonstreptococcal bacteria (NSTR).

To calculate proportional occupancy, confocal images were first binarized to discriminate between background and foreground using a custom binarization pipeline (MATLAB, Simulink). The intensity threshold was calculated with Otsu’s method using a histogram populated by all 2D slices in a 3D stack. Each channel (green, red, and magenta) from the images was binarized individually, and channels were later combined to produce a single multichannel binary image. The binary images were then analyzed using a proportional occupancy pipeline developed in R. In brief, a focal voxel in the 3D image is picked at random, and then the voxels of a specific channel that are located within a distance interval (radius 1 and radius 2) away from the focal voxel are counted. The proportional occupancy is calculated by multiplying the number of voxels within a distance range by the size of each voxel, divided by the total volume of that distance interval:

$$Proportional\;occupancy=\frac{number\;of\;voxels\;in\;distance\;interval\times voxel\;volume}{total\;volume\;of\;interval}$$

Proportional occupancy was calculated for a representative amount of focus voxels per image, starting from a distance of 1 µm away from each focal voxel, using 1 µm as a distance interval and extending up to 25 µm away from the focus voxel. Proportional occupancy was also calculated for all pairwise combinations of different focal and surrounding channels. Each channel represented *S. mutans*, *S. oralis*, or EPS.

### In Situ pH Measurement.

To assess the biofilm pH at the biofilm–apatite interface, we used a noninvasive in situ pH measurement with the fluorescent pH indicator Lysosensor yellow/blue (Molecular Probes) labeling method with multiphoton confocal microscopy and computational analysis (24). In brief, the biofilms were incubated with Lysosensor yellow/blue-labeled dextran conjugate, and the pH values within intact biofilms were measured based on fluorescence intensity ratios of the dual-wavelength fluorophore. Lysosensor yellow/blue exhibits a dual-emission spectral peak (450 and 520 nm), and the ratio between the fluorescence intensity of these two spectral peaks is pH-dependent within biofilms (24). The fluorescence intensity of both emission wavelengths and their ratio (I_450_/I_520_) within each biofilm image was measured using ImageJ 1.22. The ratios of fluorescence intensity of selected areas within each biofilm image were converted to pH values using the titration curves of ratios vs. pH (ranging from 4.0 to 7.0) as described previously (24). For pH measurement, the ratios of fluorescence intensity of selected areas within each biofilm image were converted to pH values using the titration curves and ImageJ. For visualization of in situ pH distribution at the biofilm–tooth interface, the fluorescence intensity ratios of confocal images were reconstructed using ImageJ and then rendered using Amira software. The fluorescence intensity was converted into grayscale using the Amira toolbox together with the ImageJ look-up table to correlate with the pH range of 4.0 (black) to 7.0 (white). Unlabeled biofilms were also imaged to determine whether autofluorescence of biofilm bacterial and EPS components would interfere with in situ pH analyses at the wavelengths and laser intensities used in our study; there was no interference with the measurement based on our image analysis. A detailed step-by-step protocol and procedures are provided in *SI Appendix*, *Methods*.

### Synchronized Analysis of Biofilm Architecture and Surface Demineralization.

We developed a sequential multistep method for synchronized biofilm and enamel surface imaging and realignment for structural and functional assessment related to the spatial localization of caries. In this method, we used our in vitro mixed-species biofilm model (as described above). For simultaneous mapping of the biofilm and the enamel surface underneath, wide-field images of the entire biofilm covering the enamel block surface were acquired using a stereomicroscope (Axis Zoom V16; Carl Zeiss) and a confocal microscope (Carl Zeiss) via high-resolution tile imaging. This approach allowed optical alignment of the biofilm structure formed on the entire enamel block surface with 10-µm length-scale precision. The structural organization was assessed via multilabeling approaches using species-specific fluorescent probes and EPS glucan matrix labeling. The pH at the biofilm–enamel interface was visualized and measured using a fluorescent pH mapping method and ratiometric analysis as described previously (24). Following biofilm imaging and pH mapping, the biomass was removed using an enzymatic treatment (mixture of 8.75 units of dextranase [Sigma-Aldrich] and 1.75 units of mutanase [a gift from Johnson & Johnson]) at 37 °C for 2 h, followed by water bath sonication for 4 min; this procedure was optimized to prevent structural alterations on the enamel surface integrity as determined by confocal topography and microhardness analyses (36). Then the enamel surface was assessed for demineralized areas with optical and fluorescence imaging complemented by quantitative TMR (25, 36, 40).

We also used Raman spectroscopy (Renishaw) to determine the levels of phosphate minerals on demineralized and nondemineralized areas of the tooth enamel. For Raman spectra measurements, a 785-nm laser was used to collect spectra between 0 and ∼2,000 wavenumbers (cm^−1^) with a 5-s dwell time from five repetitions at each spot. Collected spectra were corrected to zero, and solar flare spikes were removed within the Renishaw software. The peak height at 960 cm^−1^, corresponding to the phosphate ion content, was measured for each point spectrum. A detailed step-by-step protocol and procedures are provided in *SI Appendix*, *Methods*.

### Gene Expression in Biofilms.

RNA was extracted and purified using protocols optimized for oral biofilms (41). In brief, each biofilm was incubated in RNALater (Applied Biosystems) and then removed and processed for RNA extraction as described previously (41). The extracted RNA samples were purified and treated with DNaseI on a column using the Qiagen RNeasy Micro Kit (Qiagen). The RNAs were then subjected to a second DNaseI treatment with Turbo DNase (Applied Biosystems) and purified using the Qiagen RNeasy MinElute Cleanup Kit (Qiagen) to an RNA integrity number ≥9.5 as determined with a Agilent Bioanalyzer. We then performed qRT-PCR to measure the expression profiles of genes associated with *S. mutans* biofilm formation and fitness, including *gtfB*, *gtfC*, *gtfD*, *gbpC*, *atpB*, *atpD*, and *comC* (42). cDNAs were synthesized using 0.5 µg of purified RNA and the Bio-Rad iScript cDNA Synthesis Kit. The resulting cDNAs were amplified with the Bio-Rad CFX96 Real-Time PCR Detection System using previously published specific primers (42). A standard curve was used to transform the critical threshold cycle (Ct) values to relative numbers of cDNA molecules. Comparative expression was calculated by normalizing each gene of interest to the 16S rRNA, and these values were used to determine the fold change between intermixed and segregated communities.

### In Situ Gene Expression Levels within Biofilm Architecture.

To examine spatial cell organization and in situ gene expression simultaneously, an *atpB*-green fluorescent promoter (GFP) strain was used as described previously (24). In addition, in situ pH was measured and visualized as described above. The *atpB* promoter activity of *S. mutans* across biofilm 3D architecture was measured at specific locations (proximity of *S. oralis* cells within intact corona cell arrangement) and experimental conditions (intact [control] vs. disrupted corona [dextranase-treated]) using biologically independent samples. The biofilm formed on sHA either with an intact corona-like cell arrangement (buffer-treated) or with disrupted corona from dextranase treatment was sequentially scanned using the 488-nm argon laser to minimize the cross-talk between GFP and the Lysosensor. Bacterial cells were stained with Syto60 and scanned using the 633-nm He-Ne laser. The *S. oralis* corona cell arrangement was visualized using the fluorescence subtraction method, involving subtraction of the *S. mutans* GFP image stack from the total bacteria stained in the Syto60 image stack. For the corona disruption experiment, biofilms were treated with 100 units of dextranase in Na_2_HPO_4_-citric acid buffer (pH 5.0) at 37 °C for 60 min. For intact corona as a control, the biofilm was incubated in the same buffer without dextranase. After dextranase treatment, biofilms were gently dip-washed three times and then incubated in the acidic or neutral pH buffer, and confocal images were acquired after 10, 30, and 60 min of incubation (24). Similar-sized (diameter and thickness) biofilms were used for the analysis of in situ imaging. For enhanced contrast, we transformed the grayscale into a different range of colors by applying ImageJ’s lookup table (LUT) (24); the Fire LUT was applied for the visualization of *atpB* expression instead of green to avoid color overlap, since green was used to depict *S. mutans* cells, while red was used to depict *S. oralis* cells. A detailed step-by-step protocol and procedures are provided in *SI Appendix*, *Methods*.

### Assessment of Antimicrobial Tolerance.

We examined the impact of corona-like cell arrangement on antimicrobial susceptibility by viable cell counts and a time-lapse live/dead assay (30). Biofilms with an intact (enzyme buffer-treated control) or disrupted (by dextranase treatment) corona cell arrangement were prepared as described above. Viable cell counts (CFU) of *S. mutans* and *S. oralis* were recovered on blood agar plates from the biofilms with either an intact or a disrupted corona structure following topical treatment with 0.12% chlorohexidine (CHX) or vehicle (NaOAc buffer) for 5 min; CFU was normalized by dry weight. For time-lapse labeling of live and dead bacterial cells in the corona-intact or -disrupted biofilm, we used 5 µM Syto9 and 30 µM propidium iodide (Molecular Probes) in NaOAc buffer (pH 5.5). Images of the same field of view were acquired after the addition of dextranase. At 60 min, CHX (0.12% final concentration) was carefully added to the same buffer solution to determine the killing activity. Images were acquired at 5 min after the antimicrobial treatment.

### Statistical Analysis.

Data are presented as median (range) or mean ± SD and were analyzed using the Mann–Whitney *U* test (nonnormal variations in the clinical sample) or Student’s *t* test (ordinary variations in the laboratory sample) for a pairwise comparison. Data were analyzed using ANOVA with Tukey’s post hoc HSD test for a multiple comparison. Differences between groups were considered statistically significant at *P* < 0.05 unless stated otherwise. All statistical analyses were performed using SPSS version 18.0.

### Data Availability.

All pertinent data are provided in the main text and *SI Appendix*.

## Supplementary Material

Supplementary File
