# Supplementary material for: Spatial mapping of polymicrobial communities reveals a precise biogeography associated with human dental caries
Source: Proc Natl Acad Sci U S A. 2020 May 18;117(22):12375–86. doi: 10.1073/pnas.1919099117 (PMC7275741; doi:10.1073/pnas.1919099117)
Supplement: Supplementary File [file pnas.1919099117.sapp.pdf]

PNAS
www.pnas.org

Supplementary Information for

Spatial mapping of polymicrobial communities reveals a precise biogeography associated with human dental caries

D. Kim, J. P. Barraza, R. A. Arthur, A. Hara, K. Lewis, Y. Liu, E. L. Scisci, E. Hajishengallis, M. Whiteley*, H. Koo*

*Correspondence to: E-mail: koohy@upenn.edu (H.K.); mwhiteley3@gatech.edu (M.W.)

This PDF file includes:

Figures S1 to S11
Supplementary methods (step-by-step protocols and a tutorial guide)

SI Figures

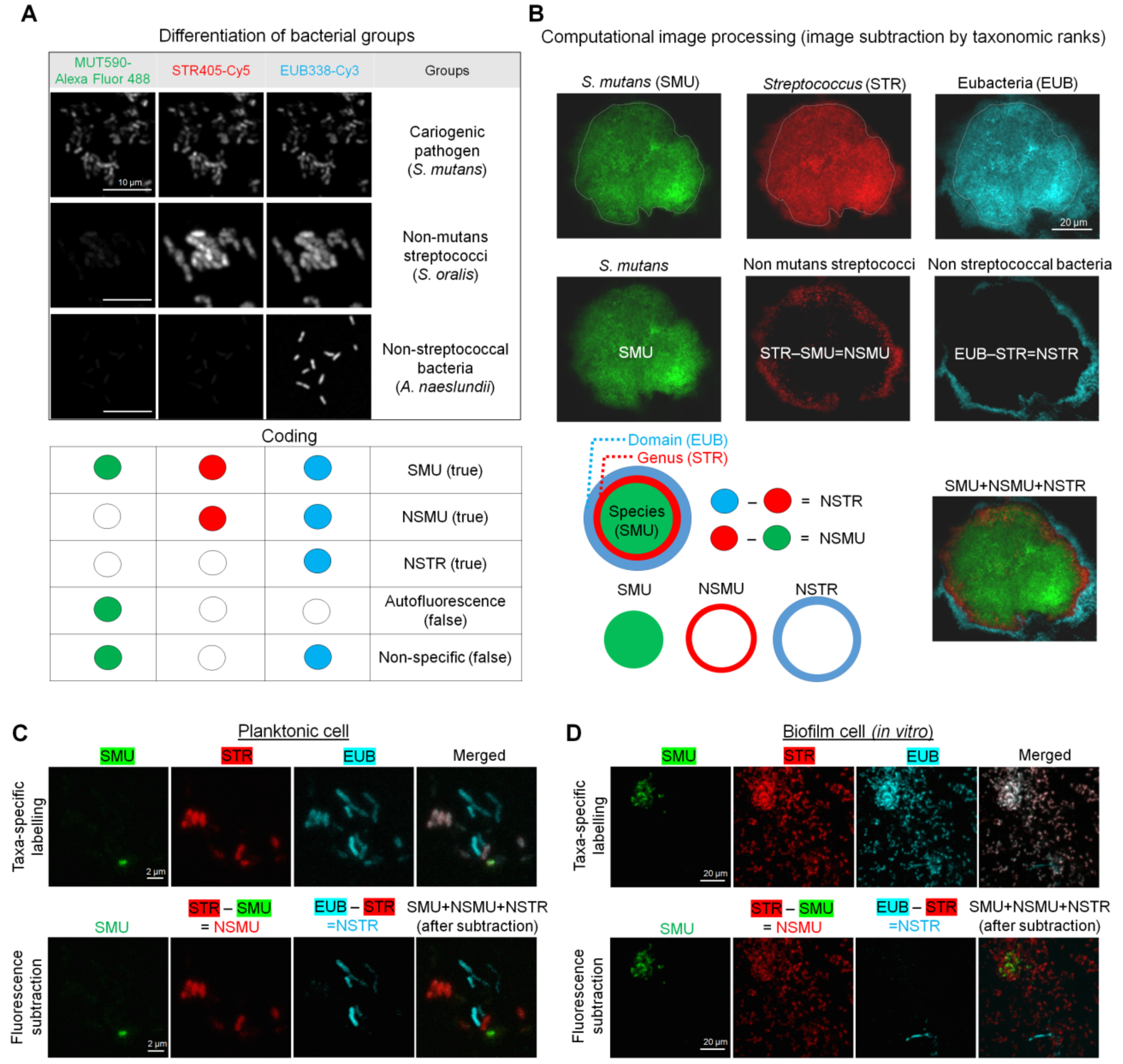

Fig. S1. Bacterial FISH probe validation and computational image processing. (**A**) Differentiation of bacterial group via taxa-specific labelling in planktonic cells. Bacterial groups were differentiated by a specific coding based on the classification of fluorescent labelling. Taxa-specific labelling was capable of separating *S. mutans*, non-mutans streptococci (NSMU) and non-streptococcal bacteria (NSTR). *S. mutans* was labelled by EUB, STR and SMU, *S. oralis* was labelled by EUB and STR, and *A. naeslundii* was labelled by EUB only. This method was able to exclude the nonspecific labelling and autofluorescence signals of clinical sample. (**B**) A fluorescence subtraction method for the configuration of polymicrobial community organization in intact plaque biofilm. For cell arrangement configuration (positioning of *S. mutans* across the microbial consortium) within the polymicrobial biofilm structure, fluorescence subtraction was applied through classification of a set of elements using Image Calculator of ImageJ: SMU, *S. mutans* alone; *Streptococcus* (STR) – SMU = non-mutans streptococci (NSMU); All bacteria (EUB) – STR = non-streptococcal bacteria (NSTR). (**C**) Application of taxa-specific labelling and fluorescence subtraction methods in planktonic cell mixture. To further demonstrate and validate this approach, we prepared planktonic cells of *S. mutans* (at OD_{600} 1.0; 2×10^9 CFU ml^{-1}), *S. oralis* (at OD_{600} 1.0; 8×10^8 CFU ml^{-1}) and *A. naeslundii* (at OD_{600} 1.5; 8×10^8 CFU ml^{-1}), each of which was labelled with taxa-specific probes and acquired image was subjected to fluorescence

subtraction method. Top images in the panel C show the taxa-specific labelling (e.g., *S. mutans* was labelled in SMU, STR, EUB). Bottom images of panel C show how fluorescence subtraction can separate non-mutans streptococci (i.e., *S. oralis*, depicted in red) and non-streptococcal bacteria (i.e., *A. naeslundii*, depicted in blue) from planktonic cell mixture via taxa-specific labelling. In the cell mixture at equal proportion of *S. mutans, S. oralis* and *A. naeslundii* (1:1:1 ratio for each of the bacterial suspension at ~10^8 CFU ml^{-1}), subtraction of SMU from STR is non-mutans streptococci (since both *S. mutans* and *S. oralis* were labelled by STR and EUB but *S. oralis* was not labelled by SMU). Next, subtraction of STR from EUB is non-streptococcal bacteria (*A. naeslundii* was labelled by EUB only while *Streptococcus*-genus (i.e., *S. mutans* and *S. oralis*) was labelled by STR and EUB). (**D**) Application of taxa-specific labelling and fluorescence subtraction methods in a mixed-species biofilm. We also prepared a mixed-species *in vitro* biofilm model to validate the fluorescence subtraction methods. Each of the bacterial suspension was mixed to provide an inoculum with a defined microbial population of *S. mutans* (10^5 CFU ml^{-1}), *S. oralis* (10^7 CFU ml^{-1}) and *A. naeslundii* (10^6 CFU ml^{-1}). The mixed population was inoculated in 2.8 ml of medium containing 1% (w/v) sucrose to form the biofilm community on the apatitic surface. Taxa-specific labelling and fluorescence subtraction were applied as described above. Details of the methodology are provided in SI Methods.

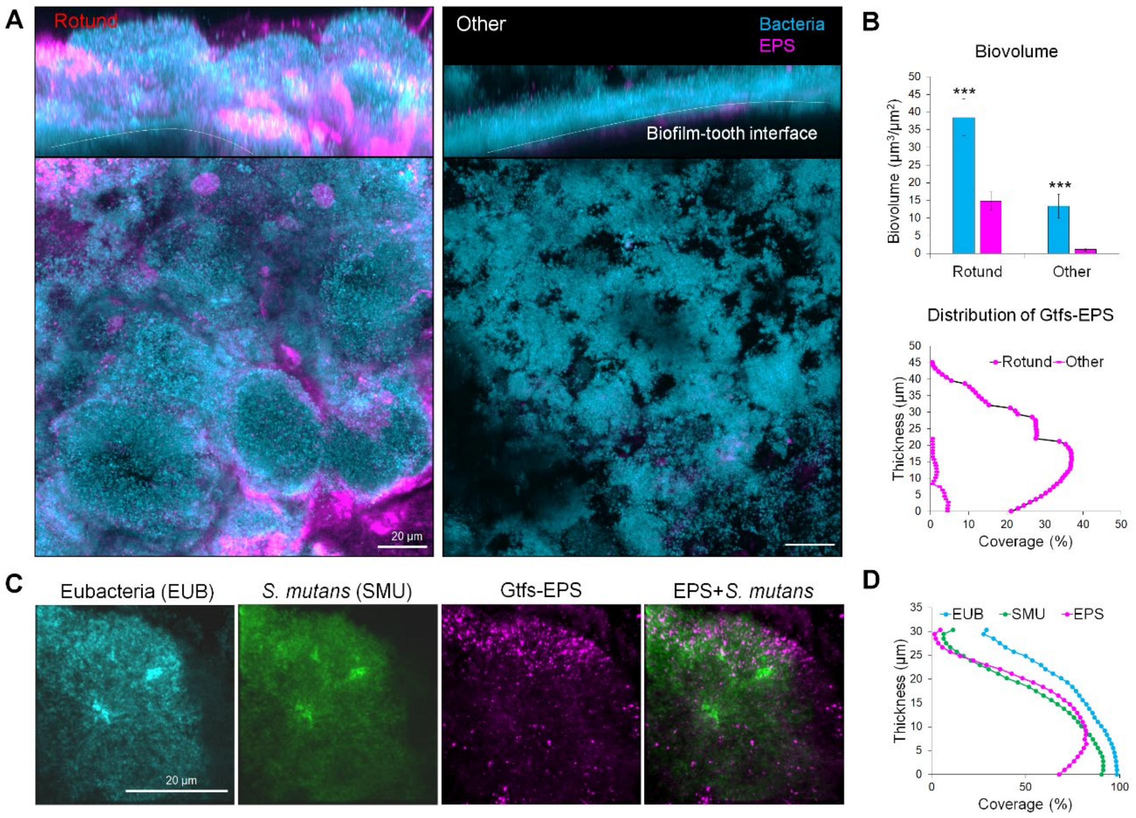

Fig. S2. Functional EPS-producing Gtfs activity across the rotund architecture in intact biofilms. (**A**) Functional Gtfs activity and EPS production within distinctive biofilm architectures. Functional Gtfs activity was measured via incorporation of fluorescently-labeled dextran into glucan (labelled with Alexa Fluor 647) in the presence of sucrose-substrate. Differential Gtf activity was observed from distinctive biofilm architectures (rotund vs. other). (**B**) COMSTAT analysis of bacterial cells and Gtfs-EPS distributions across biofilm thickness; rotund architecture displayed high levels of Gtfs-derived EPS, while the other type showed minimal Gtfs-EPS. Data are mean ± SD (n = 4). Data are mean ± SD, ****P* < 0.001 by two-tailed Student *t*-test. (**C**) The rotund architecture was dominated by *S. mutans* and Gtfs-EPS production. (**D**) Distribution of *S. mutans* cells and Gtfs-EPS across the depth indicate the bacterial cells closely associated with the EPS.

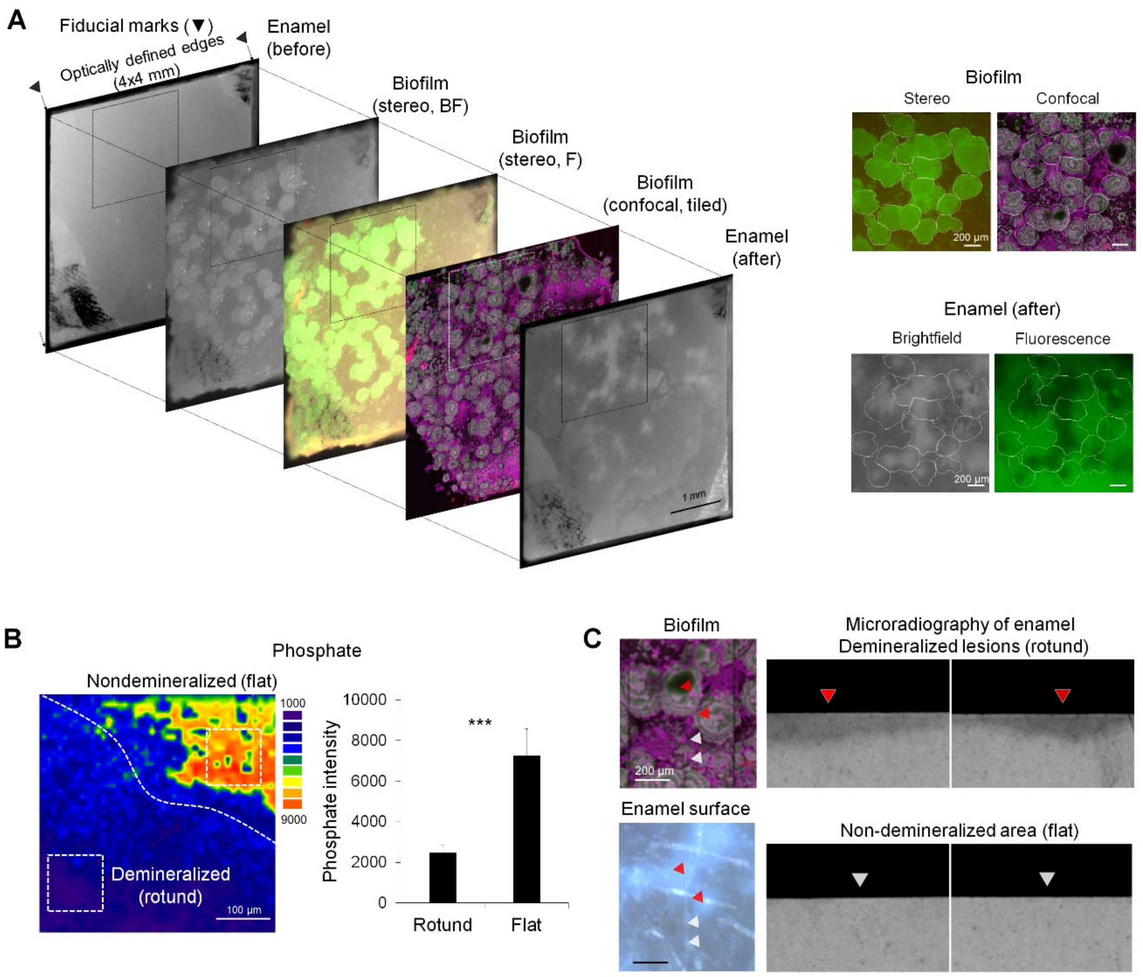

Fig. S3. Synchronized analysis of biofilm architecture and tooth-enamel surface demineralization. (**A**) Optical alignment via a hybrid confocal-stereomicroscope system to match the regions of biofilm architectures and demineralized lesions on the enamel surface. (**B**) The same enamel blocks were placed under the confocal Raman microscope and the positioning adjusted using the fiduciary marks to match the location of the biofilm structures and the demineralized areas. The distribution and content of phosphate minerals in demineralized lesions were analyzed using Raman spectroscopy as a non-destructive technique. Quantitative data were generated from the dotted-line box areas with 100 measuring points (100×100 μm^2). (**C**) Demineralized lesions associated with the biofilm rotund architecture. Red arrow heads indicate the enamel underneath rotund architecture while white arrow heads indicate the enamel covered by flat communities. Details of the methodology are provided in SI Methods.

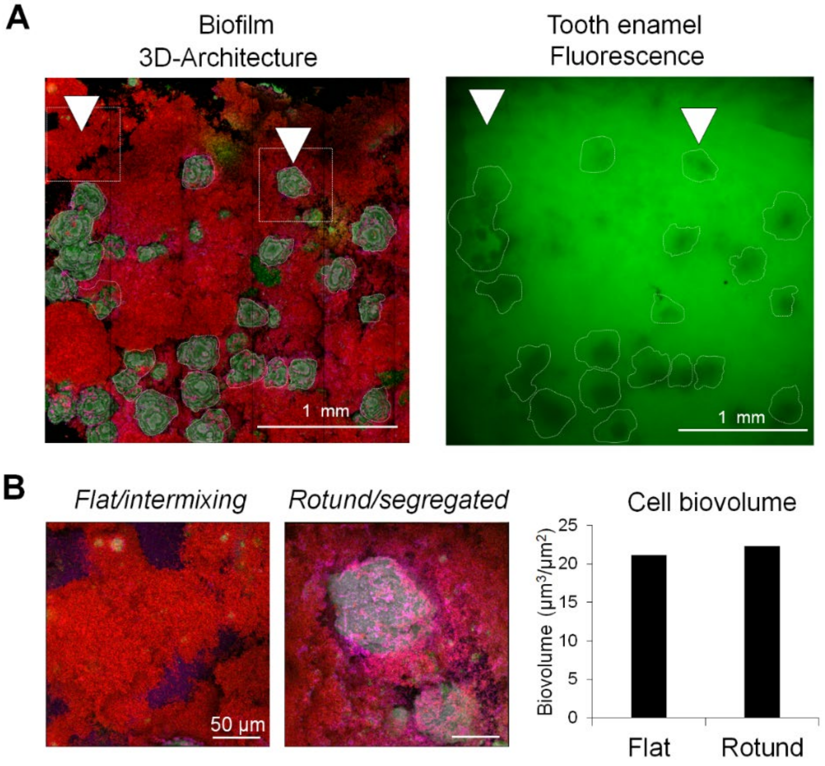

Fig. S4. Impact of bacterial biomass on demineralization. (**A**) Confocal image of biofilm architecture on enamel surface and fluorescence image of the surface underneath biofilm (after biofilm removal). Selected two areas (indicated by arrow heads) containing rotund and flat architectures (left panel A). As shown in the right panel **A** (enamel surface), the demineralized areas (which appear as localized dark spots under fluorescence imaging) matched with the positioning of the rotund architectures. (**B**) Close-up image of two distinctive (flat and rotund) structures (312×312 μm^2) and their biovolume representing the overall biomass occupied by bacterial cells (via quantitative imaging analysis (COMSTAT)); both types contained similar bacterial biomass. Details of the methodology are provided in SI Methods.

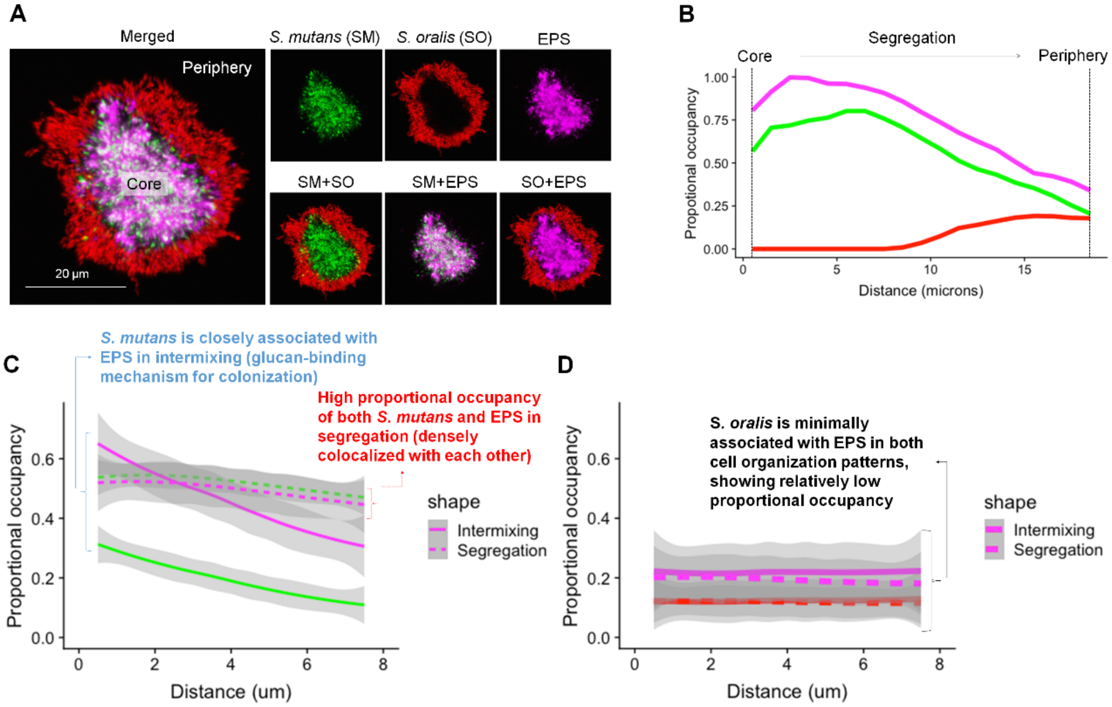

Fig. S5. Spatial organization patterns between bacteria and EPS. (**A**) Confocal images of bacteria and EPS in the segregated corona-like cell arrangement. (**B**) Proportional occupancy (PO) following the distance among biofilm components (*S. mutans*/*S. oralis*/EPS) from the *S. mutans* inner core. (**C**) Association between bacteria and EPS from intermixed or segregated cluster. *S. mutans* is spatially organized in close association with EPS matrix showing extensive co-localization of EPS glucan with the bacterium. *S. mutans* has high proportional occupancy with abundant EPS. (**D**) *S. oralis* is minimally associated with EPS, showing relatively low proportional occupancy. Shaded areas depict mean ± SD (n = 6). Details of the methodology are provided in SI Methods.

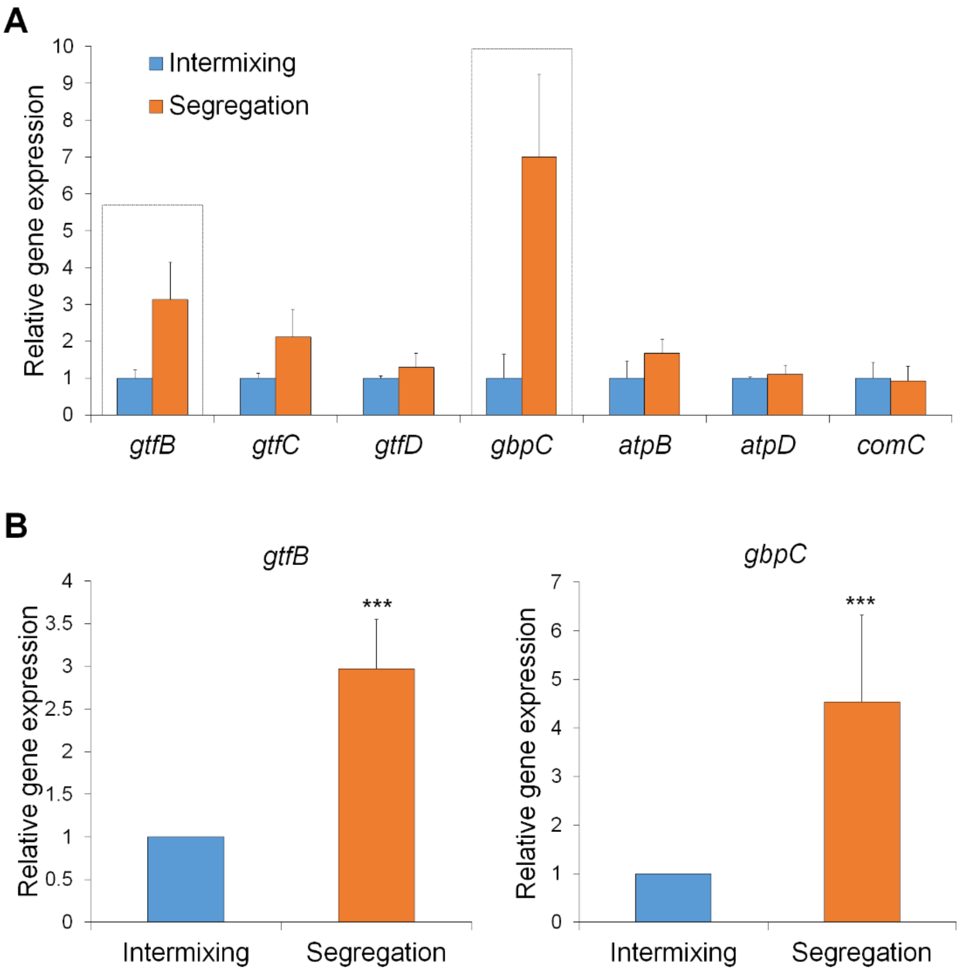

Fig. S6. Selected *S. mutans* genes expression in mixed-species biofilms. (**A**) Overall pattern of gene expression associated with biofilm formation and fitness (n = 4) between intermixed and segregated *S. mutans* cells. (**B**) The expression of *gtfB* and *gbpC* is associated with *S. mutans* virulence and biofilm structural integrity. Data are mean ± SD (n = 10), ****P* < 0.001 by two-tailed Student *t*-test.

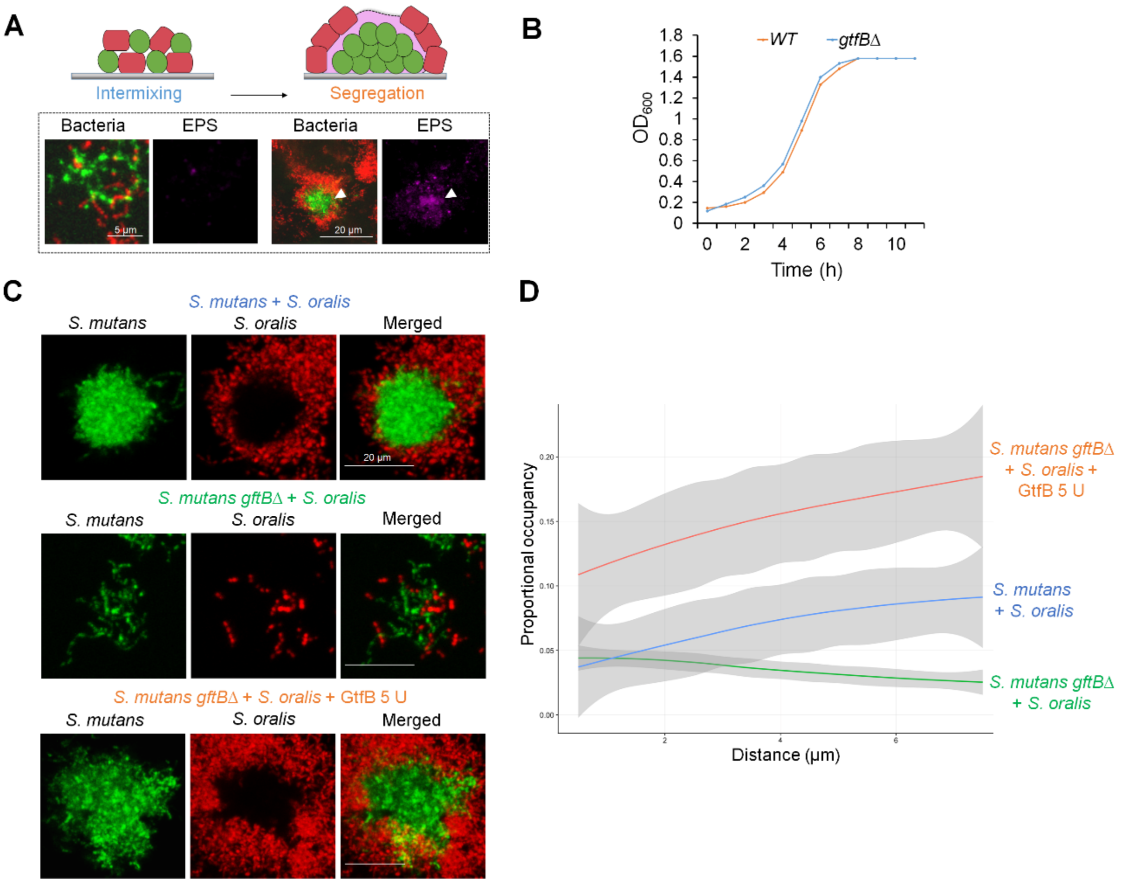

Fig. S7. GtfB-derived EPS mediates spatial cell segregation of *S. mutans* in mixed-species biofilms. (**A**) GtfB-EPS mediates *S. mutans* clustering and spatial segregation with *S. oralis* as the mixed community transition from intermixed to segregated spatial cell arrangement. (**B**) Bacterial growth curve of *S. mutans* wild type (WT) and *gtfB* mutant strains. Each bacterium was grown in ultrafiltered tryptone-yeast extract broth (UFTYE; 2.5% tryptone and 1.5% yeast extract, pH 7.0) with 1% glucose at 37°C and 5% CO_2. (**C**) Lack of GtfB-EPS in mixed-species biofilm co-cultured with *S. mutans gtfBΔ* resulted in intermixing pattern while segregated cell arrangement was re-established in mixed-species biofilm through GtfB supplementation. (**D**) Quantitative analysis for bacterial cell arrangement in the mixed biofilm. In *S. mutans* WT + *S. oralis* mixed biofilm, the proportional occupancy (PO) of *S. oralis* increased along with distance when *S. mutans* was used as a focal point (non-random distribution associated with corona-like arrangement). However, PO did not change across distance in the *gtfB* mutant + *S. oralis* mixed biofilm indicating random distribution. In the *gtfB* mutant + *S. oralis* mixed biofilm supplemented with GtfB, PO of *S. oralis* showed a similar pattern to that of mixed biofilm with *S. mutans* WT, indicating reestablishment of corona-like arrangement.

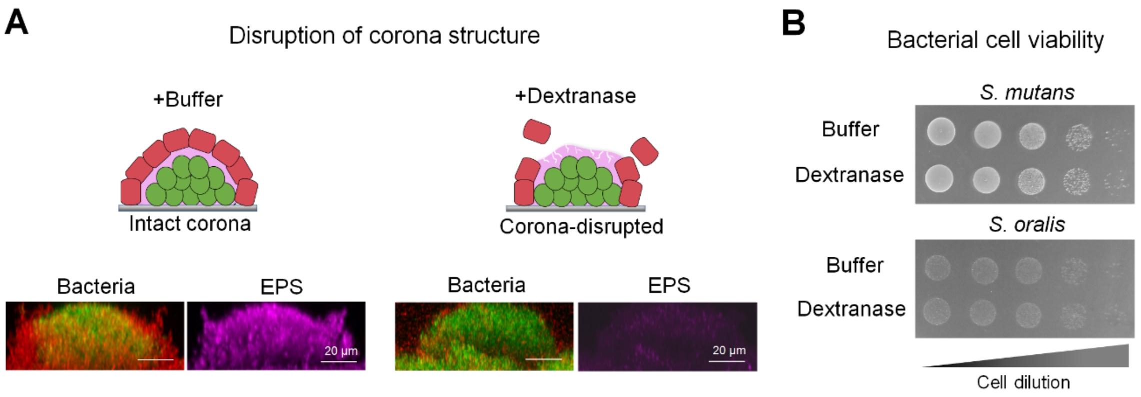

Fig. S8. Effects of dextranase on corona structure and cell viability. (**A**) Exposure of dextranase (for 60 min) resulted in degradation of the EPS matrix (magenta), which disrupted the top and outermost layer of *S. oralis* (red) without disturbing the structural integrity of the *S. mutans* (green) inner core. (**B**) Dextranase (100 units) did not affect bacterial cell viability compared to enzyme-buffer treated control.

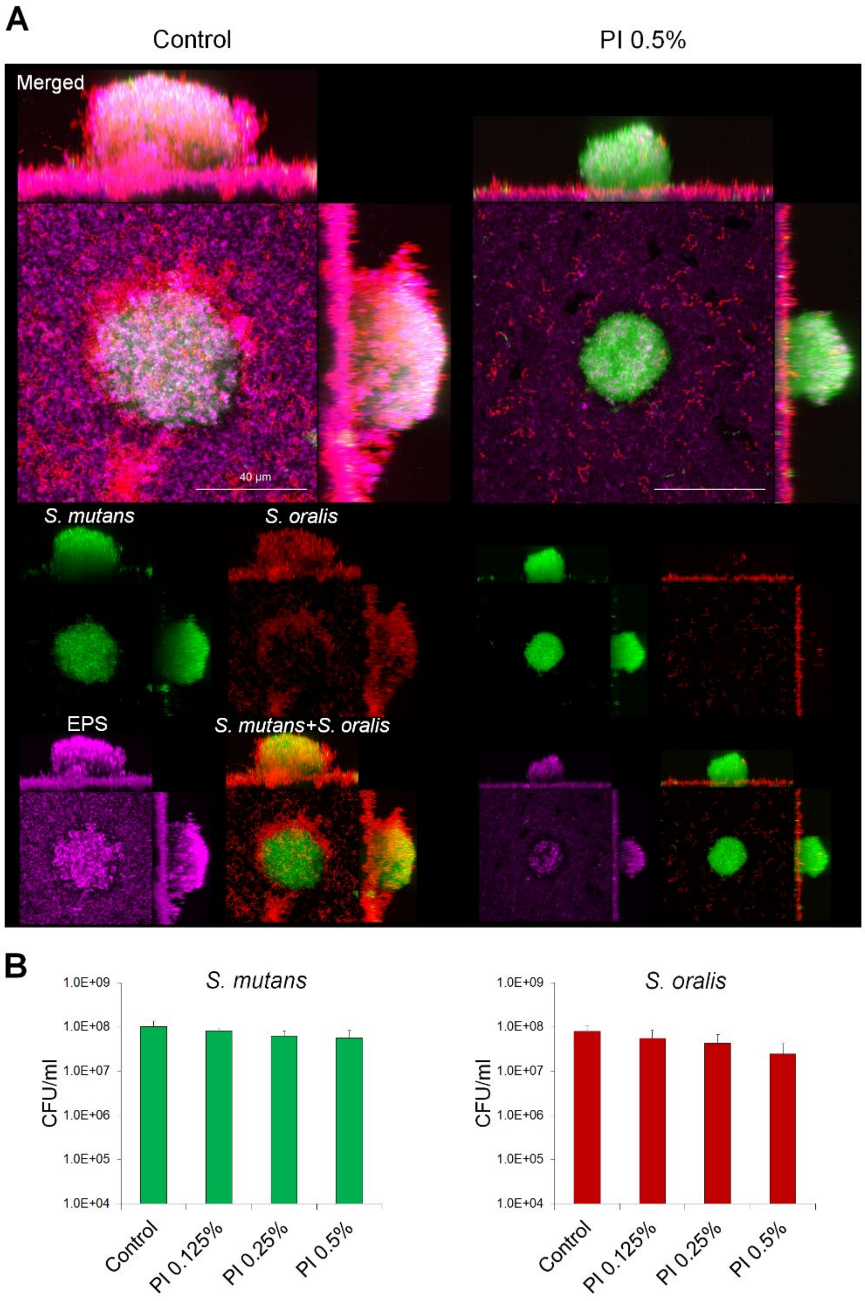

Fig. S9. Effect of Gtfs inhibitor on corona-like cell arrangement. (A) Topical treatment of povidone iodine (PI, at 0.5%) disrupted the corona structure. (B) Cell viability of *S. mutans* and *S. oralis* cells treated with PI (0–0.5%). Data are mean ± SD (n = 4). The data were subjected to analysis of variance (ANOVA) in the Tukey's HSD test for a multiple comparison. Differences between groups were considered statistically significant when $P < 0.01$. No antibacterial effects were observed with PI at concentrations between 0.125% and 0.5%.

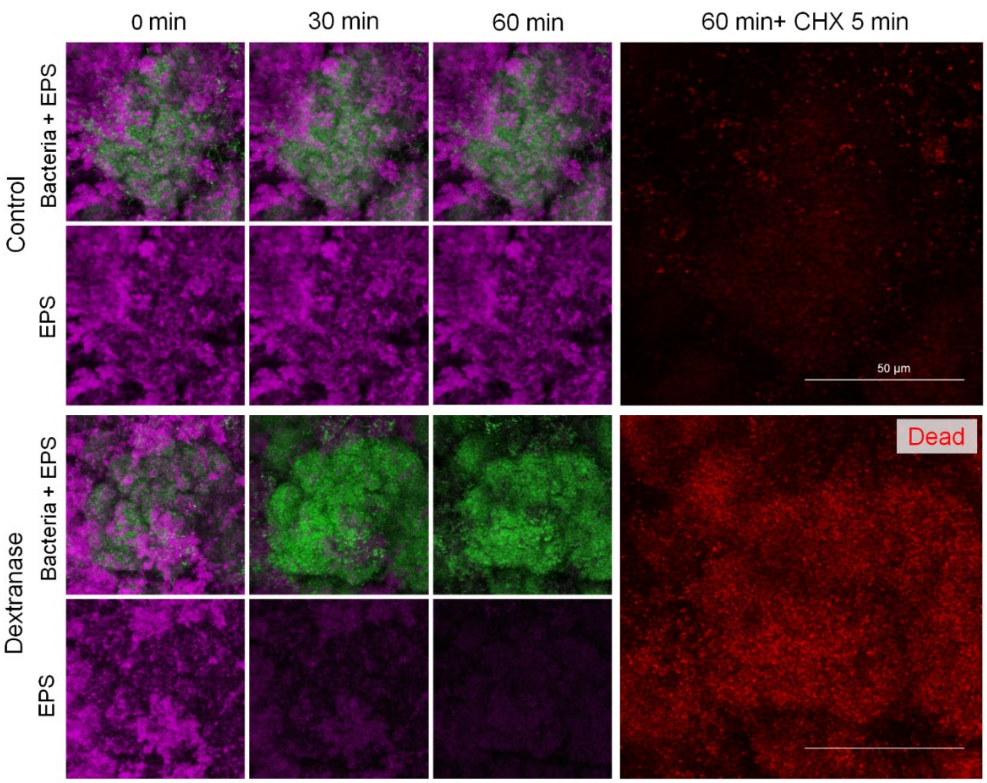

Fig. S10. Time-lapsed live and dead assay for assessment of antimicrobial susceptibility in corona-disrupted biofilm. The left panels set show time-lapsed images of biofilms treated with buffer (top panels) and dextranase (bottom panels). The buffer-treated biofilms (intact) show bacteria (in green) embedded within EPS-matrix (magenta). Dextranase-treated biofilm (disrupted) show effective degradation of the EPS matrix, disrupting the protective arrangement. The right large panels show live and dead (in red) fluorescence of biofilms (intact or disrupted) after exposure to chlorohexidine (CHX, 0.12% (v/v)) for 5 min. Disrupted biofilm display higher susceptibility (more dead cells) to killing by the antimicrobial agent (CHX) compared to intact biofilm.

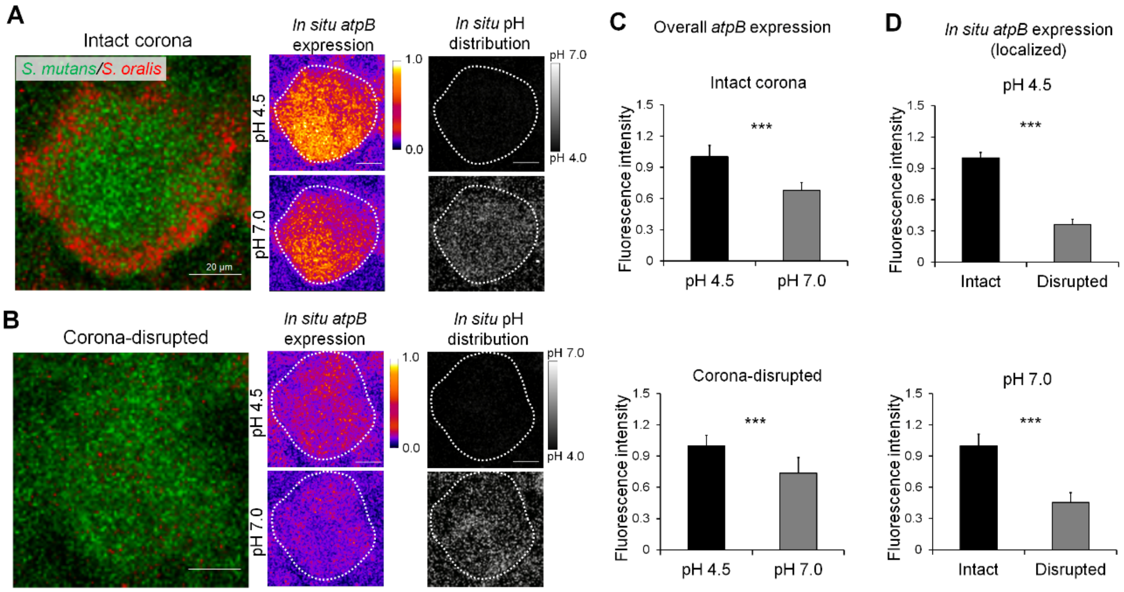

Fig. S11. *In situ atpB* gene expression of *S. mutans* within intact or disrupted corona structure. *In situ* gene expression and pH distribution in intact corona structure (buffer-treated control (without dextranase)) (**A**) and corona-disrupted (with dextranase treatment) (**B**). (**C**) The *atpB* expression at acidic pH (4.5) compared to pH 7.0 in either intact or disrupted biofilm. (**D**) Localized *in situ atpB* expression according to different pH values when comparing intact vs. disrupted biofilm. Data are mean ± SD (n = 15), ***$P < 0.001$ by two-tailed Student's *t*-test. Details of the methodology are provided in SI Methods.

SI Methods

In this section, we provided step-by-step protocols for each of the imaging methods: I) Intact human plaque biofilm imaging, II) Synchronized imaging of biofilm structure, pH and enamel surface, III) Simultaneous analysis of *in situ* gene expression and pH within biofilm

Step-by-Step Protocols

I. Intact human plaque biofilm imaging

Brief description: Imaging of undisturbed spatial structure in biofilm remains challenging as it requires sample collection from clinical sites and further processing that disrupts the original architecture. We have applied an imaging methodology to map the spatial organization of biofilm communities in its native state on extracted teeth from patients affected by severe childhood caries. We used taxa-specific fluorescent probes including *S. mutans*-specific, *Streptococcus*- and all bacteria-probes with confocal imaging across multiple length scales (from submillimeter to submicron level). Then, we used a fluorescence subtraction method (details in **Tutorial Guide 1**) to analyze the spatial arrangement and composition across different phylogenetic scale, i.e. *S. mutans* (SMU), non-mutans streptococci (NSMU), and non-streptococcal bacteria (NSTR). This multi-scale approach allowed us to study both the overall organization and spatial arrangement of the intact biofilm communities (SI methods Fig. 1).

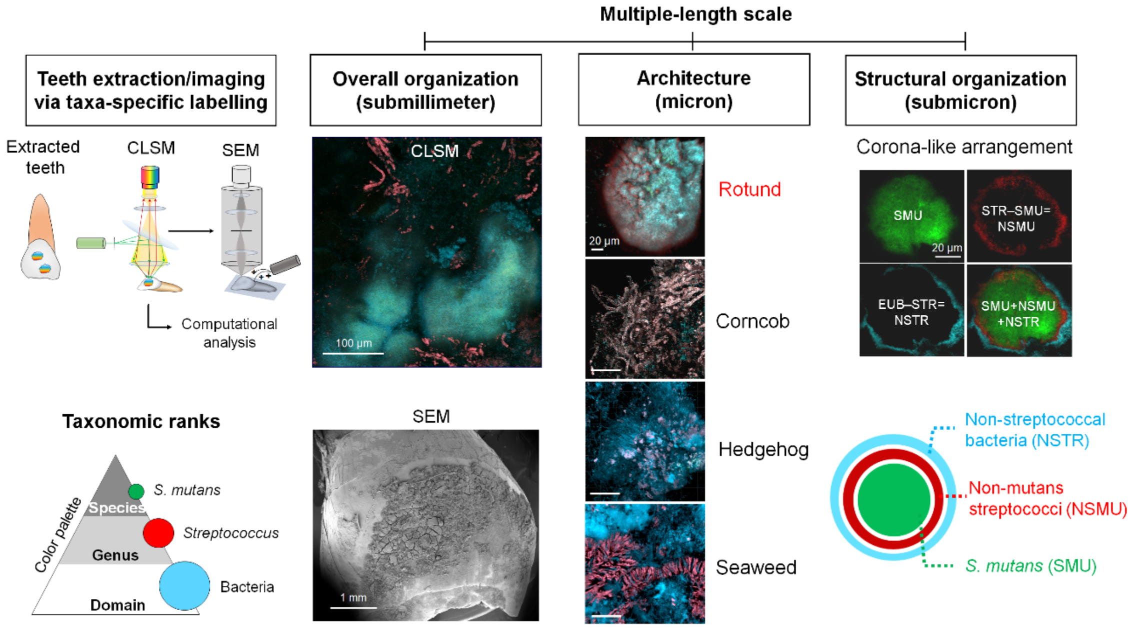

SI Methods Fig. 1. Graphical summary of the multi-scale imaging for intact biofilm on tooth surface.

Procedure:

1. Tooth samples were extracted without perturbing the naturally formed biofilms (see details in Materials and Methods), transferred to a dish (ø35×10 mm) containing sterile, PBS-soaked gauze placed at the bottom to hold the extracted teeth under wet condition (without disturbing the biofilm structure), and immediately transferred to the lab.
2. The biofilm on tooth surface was gently washed twice with PBS (pH 7.4) at room temperature, and fixed with 4% paraformaldehyde (in PBS, pH 7.4) at 4°C for 4 h.

3. After fixation, the specimen was washed twice with PBS, then transferred into 50% EtOH in PBS (pH 7.4), and stored at –20˚C.
4. For fluorescence *in situ* hybridization (FISH), the specimen was treated with lysis buffer containing 10 mg ml^{-1} lysozyme (in 20 mM Tris-HCl pH 7.5, 5 mM EDTA) for 14 min at 37°C to enhance cell permeability to the FISH probes.
5. The specimen was incubated in the hybridization solution (25% formamide, 0.9 M NaCl, 0.01% SDS, 20 mM Tris-HCl, pH 7.5) containing FISH oligonucleotide probes (MUT590, 5'-ACTCCAGACTTTCCTGAC-3' with Alexa Fluor 488 for *Streptococcus mutans*; STR405, 5'-TAGCCGTCCCTTTCTGGT-3' with Cy5 for *Streptococcus*; EUB338, 5'-GCTGCCTCCCGTAGGAGT-3' with Cy3 for all bacteria at a final concentration of 1 μM) at 46°C for 4 h, and then washed using washing buffer (0.2 M NaCl, 20 mM Tris-HCl pH 7.5, 5 mM EDTA, 0.01% SDS) at 46°C for 15 min.
6. After *in situ* hybridization, the specimen was immobilized on a small petri dish (ø35×10 mm) using a dental wax (red sprue wax) (which allowed adjustment of the position and the angle of the tooth surface to be in a parallel plane in relation to the confocal microscope objective).
7. Biofilm images were acquired using confocal microscopy (LSM 800 (Zeiss) equipped with a 20× (1.0 numerical aperture (NA)) water immersion objective. The field of view was adjusted using fine focus knob under green fluorescence or refection mode.
8. The biofilms were sequentially scanned using Diode lasers (488, 561 and 640 nm), and the fluorescence emitted was collected with the GaAsP or multialkali PMT detector (490–550 nm for Alexa Fluor 488 (*S. mutans*), 565–620 nm for Cy3 (all bacteria), and 645–700 nm for Cy5 (*Streptococcus*).
9. Initially, low magnification (0.5×zoom) (submillimeter-scale: 0.624×0.624 (x,y) mm^2) images were acquired. Then, high magnification (1×zoom, 312×312 $μm^2$; 2×zoom, 156×156 $μm^2$; 4×zoom, 78×78 $μm^2$) images were further acquired for biofilm architecture (micron-scale).
10. For quantitative analysis, we selected each of the architectures in a field of view at 312x312 $μm^2$, and optical sectioning with a z-stack size of 0.92 μm.
11. The confocal images were analyzed using COMSTAT (available as free download at http://www.imageanalysis.dk), written as scripts for MATLAB software to calculate the biovolume of each architecture (details in refs (1, 2)).
12. Total biovolume was measured from EUB, STR and SMU-labelled cells. The biovolume of EUB and SMU-labelled cells were further used for measuring a relative ratio of SMU to EUB.
13. Following overall biovolume quantification, we employed a fluorescence subtraction method to analyze the spatial arrangement and composition across different phylogenetic scale, i.e. *S. mutans* (SMU), non-mutans streptococci (NSMU), and non-streptococcal bacteria (NSTR) **(see Tutorial Guide 1)**.
14. After confocal imaging, the same specimen was used to acquire electron micrographs via scanning electron microscopy (SEM).
15. For SEM imaging, the biofilm sample was treated with 2% paraformaldeyhyde/2% glutaraldehyde for 18 h. The sample was gently submerged into the fixative solution, and subsequently washed with PBS.
16. Then, the specimen was serially treated with different concentrations of EtOH (in serial dehydration steps; 50→70→80→90→100%). The sample was kept at each concentration of EtOH for 10 min.
17. Next, the specimen was dried using hexamethyldisilazane (HMDS) (serially treated with EtOH:HMDS at 1:1 ratio, then at 1:4 ratio, and finally exposed to 100% HMDS). The sample was further dried in the fume-hood for 10 min.
18. The dried specimen was mounted on a SEM aluminum-holder with super-glue and painted with silver-liquid at the interface between teeth and holder surface.
19. The specimen was subjected to sputter coating (Au/Pd).
20. The image was acquired using a high-resolution scanning electron microscope (SEM; Quanta 250 FEG eSEM, FEI).

II. Synchronized imaging of biofilm structure, pH and enamel surface

Brief description: We developed a sequential multi-step method for synchronized biofilm and enamel surface imaging and realignment for structural and functional assessment related to spatial localization of caries. To perform this method, we used our *in vitro* mixed-species biofilm model in which *S. mutans* (pathogen) and *S. oralis* (commensal) were inoculated on natural human tooth-enamel and allowed to form a biofilm in the presence of sucrose. Following biofilm formation, we employed a hybrid confocal-stereoscope system using tiled image acquisition to encompass the entire biofilm and enamel surface. This approach allowed optical alignment of the biofilm structure formed on the entire enamel block surface with 10 μm length-scale precision. The structural organization was assessed *via* multi-labelling approaches using species-specific fluorescent probes and EPS glucan matrix labelling. The pH at the biofilm-enamel interface was visualized and measured using a fluorescent pH mapping method and ratiometric analysis as detailed previously (2); the link for the step-by-step protocol can be found here (https://doi.org/10.1371/journal.ppat.1002623.s006). The enamel surface was assessed for demineralized areas with optical and fluorescence imaging complemented by quantitative transverse microradiography (TMR). This procedure is summarized in SI Methods Figure 2A, and used to match the biofilm architectural features with pH mapping and enamel demineralization.

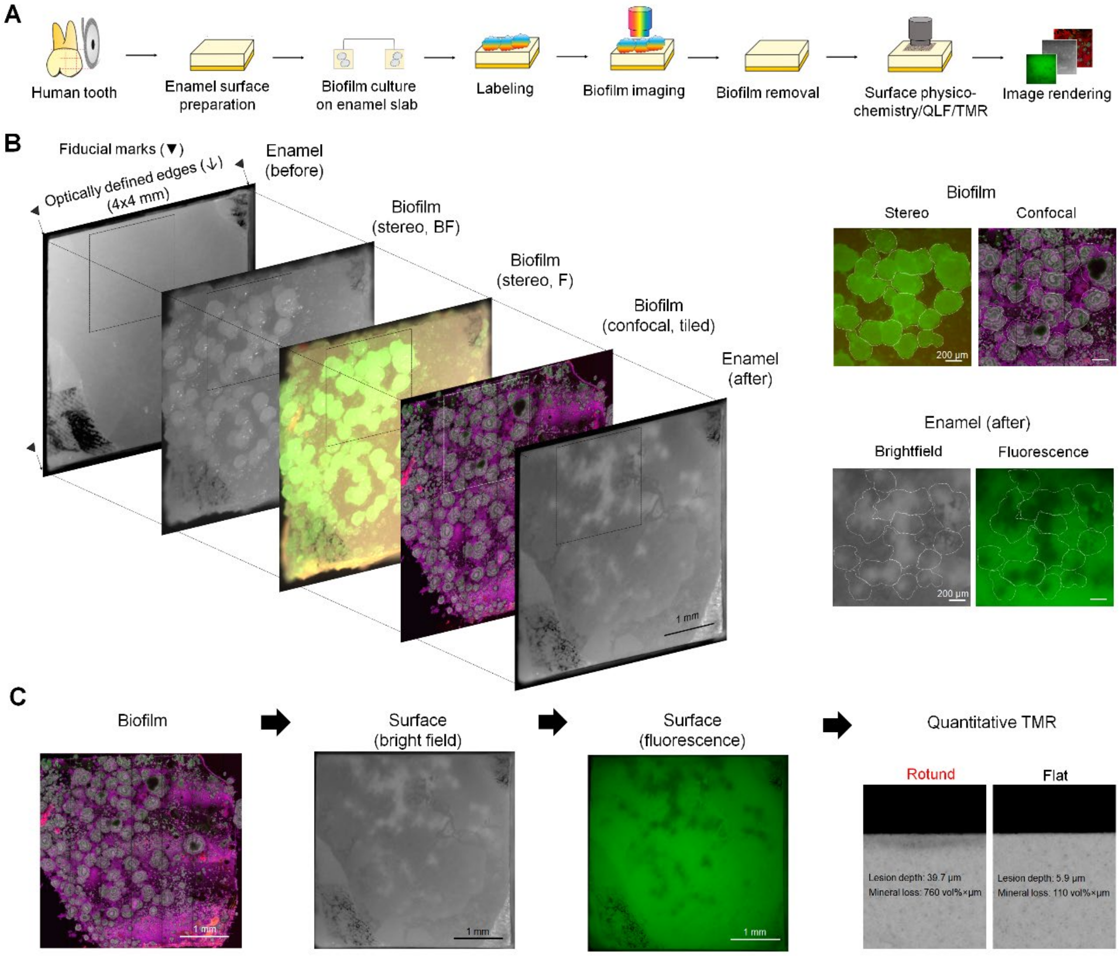

SI Methods Fig. 2. Synchronized biofilm-surface analysis using *in situ* human enamel model and multi-step imaging.

Procedure:

1. Human enamel blocks were prepared to have a uniform size (4x4 mm^2) with distinctive edges as fiduciary marks (SI Methods Fig. 2B) to facilitate optical alignment.
2. Prior to bacterial inoculation for biofilm growth, wide-field image of the entire enamel surface was acquired using a stereomicroscope (Axis Zoom V16, Zeiss) equipped with a 1× objective (PlanNeoFluar Z, Zeiss) under bright field and green fluorescence (**see Tutorial Guide 2**).
3. Then, a mixed-species biofilm was prepared using saliva-coated human enamel blocks (SI Methods Fig. 2A) using the cariogenic pathogen *S. mutans* and the commensal streptococci *S. oralis*.
4. For inoculum preparation, each bacterium was grown in ultrafiltered tryptone-yeast extract broth (UFTYE; 2.5% tryptone and 1.5% yeast extract, pH 7.0) with 1% glucose at 37°C and 5% CO_2 to OD_{600} 1.0 (exponential growth phase).
5. Each of the bacterial suspension was mixed to provide an inoculum with a defined microbial population of *S. mutans* (10^5 CFU ml^{-1}) *and S. oralis* (10^7 CFU ml^{-1}).
6. The mixed population was inoculated in 1 ml of UFTYE containing 0.1% (w/v) sucrose and incubated for 19 h to form an initial biofilm community on the enamel surface.
7. Then, the biofilms formed on enamel blocks were transferred to UFTYE containing 1% sucrose to induce environmental changes to simulate a cariogenic challenge at 19 h. The culture medium was changed twice daily until the end of the experimental period (91 h).
8. For biofilm structure, EPS was labelled with 1 μM dextran-conjugated Alexa Fluor 488 and bacterial cells were labelled with 10 μM Syto60 (total) or species-specific FISH probes (MUT590, 5'-ACTCCAGACTTTCCTGAC-3' with Cy5 for *S. mutans*; MIT588, 5'-ACAGCCTTTAACTTCAGACTTATCTAA-3' with Cy3 for *S. oralis* each probe at a final concentration of 1 μM) as described in Materials & Methods and in step-by-step protocol I. For pH mapping, 1 μM of Lysosensor yellow/blue dextran conjugate was used as detailed previously (2).
9. After biofilm formation, the wide-field images of entire biofilm formed the enamel block (4x4 mm^2) were acquired using stereomicroscope (Axis Zoom V16, Zeiss) for optical alignment of biofilm-surface (**Tutorial Guide 2**). Then, high resolution images were acquired using confocal microscopy (LSM 800 (Zeiss) or TCS SP8 (Leica)) equipped with a 20× (1.0 numerical aperture (NA)) water immersion objective.
10. For biofilm structure, the biofilms were sequentially scanned using Diode lasers (488, 561 and 640 nm), and the fluorescence emitted was collected with the GaAsP or multialkali PMT detector (490–550 nm for Alexa Fluor 488 (EPS), 565–620 nm for Cy3 (*S. oralis*), and 645–700 nm for Cy5 (*S. mutans*).
11. Tile acquisition mode (entire surface 4.396×4.396 mm^2) was applied for entire biofilm imaging. Then, image stacks of the area of interest were acquired with optical zoom (1×zoom, 312x312 $μm^2$; 2×zoom, 156x156 $μm^2$; 4×zoom, 78x78 $μm^2$).
12. For pH mapping, the biofilm was scanned using two-photon confocal microscopy for dual emission (450 and 520 nm).The pH values within intact biofilms were measured based on fluorescence intensity ratios of the dual-wavelength Lysosensor fluorophore using the titration curves of ratios versus pH (ranging from 4.0 to 7.0) as described in a published protocol (https://doi.org/10.1371/journal.ppat.1002623.s006).
13. Zen Blue (Zeiss) and Amira 5.4.1 (Visage Imaging) software were used to create 3D renderings to visualize the overall architecture of the biofilms.
14. After biofilm imaging using the hybrid confocal-stereomicroscopy system, biomass was removed with an enzymatic treatment (mixture of 8.75 units of dextranase and 1.75 units of mutanase) at 37°C for 2 h followed by water-bath sonication (for 4 min).
15. The cleaned enamel surface was used for optical and fluorescence imaging via stereomicroscope (Zeiss).
16. Optically defined edges of each enamel blocks (which have distinctive edges as fiduciary marks) were used for image alignment.

17. All acquired images (before and after biofilm removal) were realigned using the edge of enamel blocks. To match community structures (e.g., rotund) and demineralized enamel lesions underneath the biofilm, acquired images were aligned using a grid box (**See Tutorial Guide 2**).
18. After image realignment, lesion depth of the demineralized enamel regions was determined using transversal microradiography (TMR).
19. For TMR, the enamel blocks were cross-sectioned using a hard tissue microtome (Scientific Fabrications Laboratories).
20. Prior to sectioning, the region of interest was selected based on the realigned biofilm-surface images (SI Methods Fig. 2C). For example, the entire cross-section of the enamel block encompassing both demineralized and non-demineralized areas was selected (see **Tutorial Guide 2**, SI Methods Fig. 14)
21. The enamel block was mounted on the microtome holder and sectioned transversally across the entire length of the enamel block to obtain a 100-µm thick section and section with a hard tissue microtome.
22. The 100-µm section was mounted on X-ray sensitive plates (Microchrome Technology) along with an aluminum calibration step wedge. The plates were developed according to the manufacturer's instructions.
23. Section was subjected to Ni-filtered Cu-Kα radiation (X-ray; Philips Electronic Instruments) at 30 mA and 20 kV for 65 min.
24. Microradiographic images were analyzed with dedicated software (TMR 2000, Inspektor) with sound enamel defined at 87% mineral volume to obtain mean lesion depth (µm).

II. Simultaneous *in situ* gene expression and pH mapping

Brief description: We developed a method for simultaneous pH mapping and *in situ atpB* expression (a key gene associated with acid tolerance and fitness) across the biofilm structure. To achieve this, we used an *atpB*-green fluorescent promoter (GFP) *S. mutans* construct combined with fluorescence pH mapping approach as detailed previously (3). The *atpB* promoter activity was measured within intact (control, buffer-treated) and disrupted (dextranase treated) corona structure (SI Methods Figure 3).

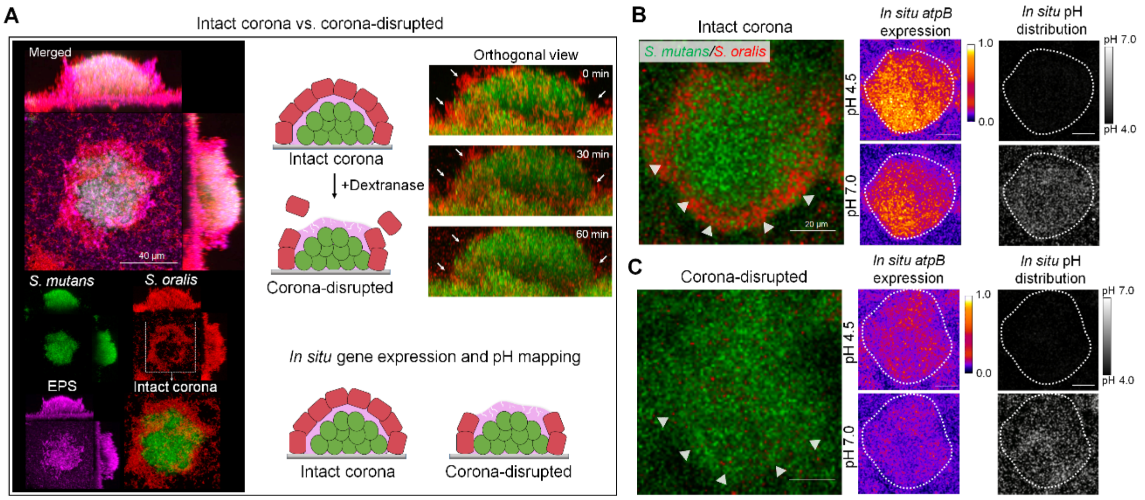

SI Methods Fig. 3. Simultaneous *in situ* gene expression and pH mapping of intact corona and corona-disrupted biofilm.

Procedure:

1. An *atpB* promoter-GFP construct (P*atpB*::gfp) of *S. mutans* UA159 was used for *in situ* gene expression (details of the construct can be found in (3)).
2. The mixed-species biofilms were formed on saliva-coated hydroxyapatite discs (surface area, 2.7 ± 0.2 cm^2; Clarkson Chromatography Inc.) as described in the step-by-step protocol II.
3. Each disc was inoculated with *S. mutans* (10^5 CFU ml^{-1}) *and S. oralis* (10^7 CFU ml^{-1}), and then incubated with Lysosensor yellow/blue dextran conjugate for pH mapping. The pH values within biofilms were measured based on fluorescence intensity ratios of the dual-wavelength Lysosensor fluorophore (see below).
4. The *atpB* promoter activity of *S. mutans* across biofilm architecture was measured at specific locations (i.e. in close proximity of *S. oralis* layer) within intact or disrupted corona structure. Disruption of the *S. oralis* corona can be achieved with dextranase treatment without disturbing the structural integrity of the *S. mutans* inner core and its cell viability (SI Methods Fig. 3A; details in procedure #9), Hence, we compared the *atpB* expression in intact (enzyme buffer-treated control) vs. disrupted corona (dextranase-treated)
5. The biofilm formed on sHA with intact corona-like cell arrangement (buffer-treated control) was incubated in Na_2HPO_4-citric acid buffer (pH 5.0) without dextranase at 37˚C for 60 min. After buffer treatment (at pH 5.0), biofilms were gently dip-washed three times and incubated in the acidic pH buffer, and the confocal images acquired after 10, 30 and 60 min of incubation. Then, the biofilm was incubated in the neutral pH buffer (pH 7.0) and the confocal images acquired after 10, 30 and 60 min of incubation.
6. The confocal images of Lysosensor-incorporated biofilms were acquired using a multi-photon laser scanning microscope (TCS SP8, Leica) equipped with a 20× (1.0 NA) water immersion lens. The biofilms were excited at 700 nm, and emission was detected in two channels: one at 450 nm (using non-descanned detector NDD1; −495 nm) and the other at 521 nm (using non-descanned detector NDD2; 495–560 nm). Bacterial cells were stained with SYTO60 and scanned using 633 nm He-Ne laser.
7. For pH measurement, the ratios of fluorescence intensity of selected areas within each biofilm image were converted to pH value. The fluorophore exhibits a dual-emission spectral peak (fluorescence emission maxima 452 nm and 521 nm), and the ratio between the fluorescence intensity of these two spectral peak is pH-dependent within biofilms as described in a published protocol (https://doi.org/10.1371/journal.ppat.1002623.s006). For visualization of pH distribution in the biofilm, fluorescence intensity ratios (corresponding to the pH values between 7.0 and 4.0) of all confocal images were reconstructed using Image J, then Amira. The fluorescence intensity was converted into grayscale using the Amira tool-box to correlate with the pH range from 7.0 (white) to 4.0 (black).
8. For measurement of *atpB* promotor activity (GFP expression) with intact corona structure, biofilm was sequentially scanned using the 488 nm Argon laser to minimize the crosstalk between GFP (515–545 nm) and Lysosensor (no signal emitted), and the fluorescence emitted was collected with the internal spectral detectors (515–545 nm). For visualization of gene expression level, we applied ImageJ's lookup table (LUT) Fire (Fire LUT) as described previously (3). Fire LUT was used for *in situ atpB* expression, while green and red were used for *S. mutans* and *S. oralis* respectively (SI Methods Fig. 3B and C).
9. The same imaging procedures were employed for corona-disrupted biofilms using dextranase. Briefly, biofilms were treated with 100 units of dextranase in Na_2HPO_4-citric acid buffer (pH 5.0) at 37˚C for 60 min (SI Methods Fig. 3A). After dextranase treatment (at pH 5.0 of buffer), biofilms were gently dip-washed three times and incubated in the acidic pH buffer, and the confocal images acquired after 10, 30 and 60 min of incubation. Then, the biofilm was incubated in the neutral pH buffer (pH 7.0) and the confocal images acquired after 10, 30 and 60 min of incubation. *In situ* pH and *atpB* promoter activity within the biofilms were simultaneously imaged as described above (procedure #7–9).
10. Quantification of signal intensity of selected area of interest was measured using ImageJ as follows; *Analyze→Tool→ROI Manager*. For comparison between intact corona and

corona-disrupted, similar size (diameter and thickness) of the biofilm structures were selected.

11. To visualize the *S. oralis* corona cell arrangement, fluorescence subtraction method was applied as follows: *total bacteria stained by SYTO60 image stack – S. mutans GFP image stack* = *S. oralis*.

Tutorial Guide

1. Fluorescence subtraction method

1.1. Taxa-specific labelling using FISH

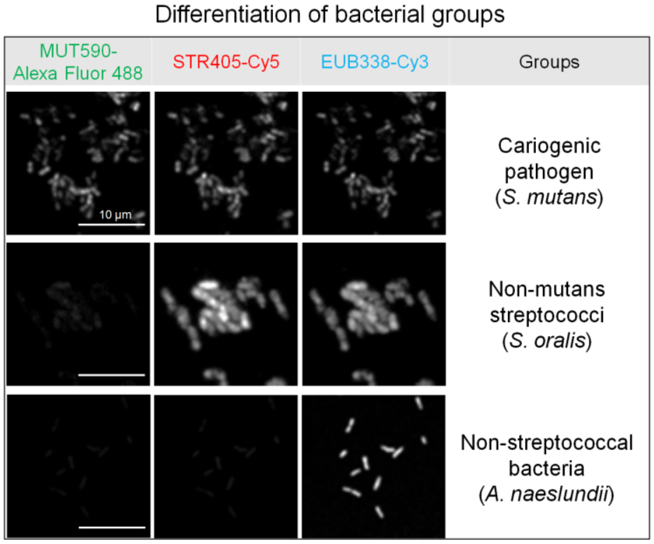

SI Methods Fig. 4. Differentiation of bacterial group via taxa-specific labelling in planktonic cells.

1.1.1. Prepare actively growing bacterial cells (exponential phase) of *Streptococcus mutans* UA159 (ATCC 700610), *Streptococcus oralis* (ATCC 35037), and *Actinomyces naeslundii* (ATCC 12140) in UFTYE containing 1% glucose (*S. mutans* (at OD_{600} 1.0; 2×10^{9} CFU ml^{-1}), *S. oralis* (at OD_{600} 1.0; 8×10^{8} CFU ml^{-1}) and *A. naeslundii* (at OD_{600} 1.5; 8×10^{8} CFU ml^{-1})).

1.1.2. Transfer 500 µl of each cell suspension to 1.5 ml tube, and centrifuge at 13000×*g* for 10 min, at 4°C

1.1.3. Wash the cell with PBS and collect the cell pellet via centrifugation (13000×*g* at 4°C).

1.1.4. Fix with 4% paraformaldehyde at 4°C for 4 h.

1.1.5. Wash the cell with PBS and keep in 50% EtOH at –20°C.

1.1.6. Remove 50% EtOH (after centrifugation) and wash with PBS.

1.1.7. Treat with lysis buffer (10 mg ml^{-1} lysozyme in 20 mM Tris-HCl pH 7.5, 5 mM EDTA) for 14 min.

1.1.8. Wash the cell pellet with PBS twice (after centrifugation).

1.1.9. For taxa-specific labelling, *in situ* hybridization (FISH) probes (16S rRNA-targeted) including all bacteria (EUB), *Streptococcus* (STR), *S. mutans* (SMU) following the taxonomic ranks (domain-genus-species) are used.

1.1.10. Incubate in the hybridization solution (25% formamide, 0.9 M NaCl, 0.01% SDS, 20 mM Tris-HCl, pH 7.5) containing FISH oligonucleotide probes (MUT590, 5'-ACTCCAGACTTTCCTGAC-3' with Alexa Fluor 488 for *S. mutans*; STR405, 5'-TAGCCGTCCCTTTCTGGT-3' with Cy5 for *Streptococcus*; EUB338, 5'-GCTGCCTCCCGTAGGAGT-3' with Cy3 for all bacteria at a final concentration of 1 µM) at 46°C for 4 h.

1.1.11. Wash the cell pellet using washing buffer (0.2 M NaCl, 20 mM Tris-HCl pH 7.5, 5 mM EDTA, 0.01% SDS) at 46°C for 15 min.

1.1.12. Wash with PBS twice and resuspend in 100 µl PBS. Put 10 µl aliquot of fluorescently labelled cell suspension on slide glass and cover with a cover glass.

1.1.13. Acquire images of planktonic cell mixture using confocal microscopy (LSM 800 (Zeiss)) equipped with a 40× (1.2 numerical aperture (NA)) oil immersion objective.

1.1.14. The planktonic cell mixture is sequentially scanned using Diode lasers (488, 561 and 640 nm), and the fluorescence emitted is collected with the GaAsP or multialkali PMT detector (490–550 nm for Alexa Fluor 488 (*S. mutans*), 565–620 nm for Cy3 (all bacteria), and 645–700 nm for Cy5 (*Streptococcus*).

1.1.15. Figure 4 shows the taxa-specific labelling of each bacterial cell. *S. mutans* is labelled by EUB, STR and SMU, *S. oralis* is labelled by EUB and STR, and *A. naeslundii* is labelled by EUB only (SI Methods Fig. 4).

1.2. Fluorescence subtraction

1.2.1. <u>Planktonic cell mixture</u>

1.2.1.1. Prepare a planktonic cell mixture at equal proportion of *S. mutans, S. oralis* and *A. naeslundii* (1:1:1 ratio for each of the bacterial suspension at ~10^8 CFU ml^{-1}).

1.2.1.2. Label the cell mixture with taxa-specific probes (following fixation-lysis-hybridization-washing steps as described above (section from 1.1.3 to 1.1.12).

1.2.1.3. Acquire image with similar signal intensity (signal intensity must be checked using confocal microscope software or ImageJ).

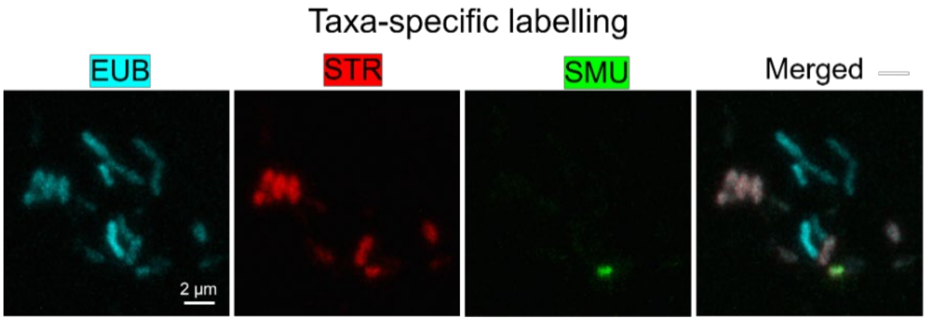

SI Methods Fig. 5. Taxa-specific labelling of planktonic cell mixture.

1.2.1.4. To analyze the composition of bacterial cells across different phylogenetic scale, i.e. *S. mutans* (SMU), non-mutans streptococci (NSMU), and non-streptococcal bacteria (NSTR), a fluorescence subtraction method is applied after taxa-specific labelling.

1.2.1.5. Computational image processing (fluorescence subtraction) is conducted using ImageJ (<u>https://imagej.net/Downloads</u> and tutorial is available at <u>https://imagej.net/Category:Tutorials</u>). Briefly, the stepwise process is described below:

1.2.1.6. <u>Step 1</u>: Open each of image stack using ImageJ; *File→Import→Image Sequence*.

1.2.1.7. <u>Step 2</u>: Check the signal intensity of each image stack using ImageJ; *Analyze→Tool→ROI Manager*. *During the confocal imaging acquisition, signal intensity of each channel is monitored by Zen Blue software.

1.2.1.8. <u>Step 3</u>: Subtract STR image stack from EUB image stack using ImageJ; *Process→Image calculator* (SI Methods Fig. 6). *Image 1, EUB; Operation, Subtract; Image 2, STR* (EUB – STR = non-streptococcal bacteria (NSTR)).

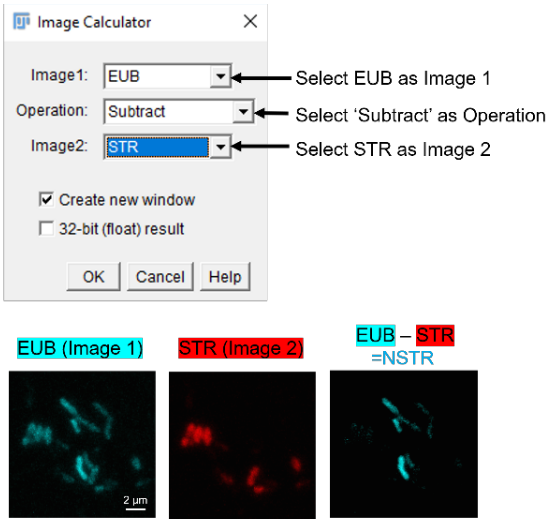

SI Methods Fig. 6. Subtraction of STR (*Streptococcus*) from EUB (all bacteria).

1.2.1.9. Step 4: Subtract SMU image stack from STR image stack using ImageJ; *Process→Image calculator* (SI Methods Fig. 7). *Image 1, STR; Operation, Subtract*; *Image 2, SMU* (STR – SMU = non-mutans streptococci (NSMU)).

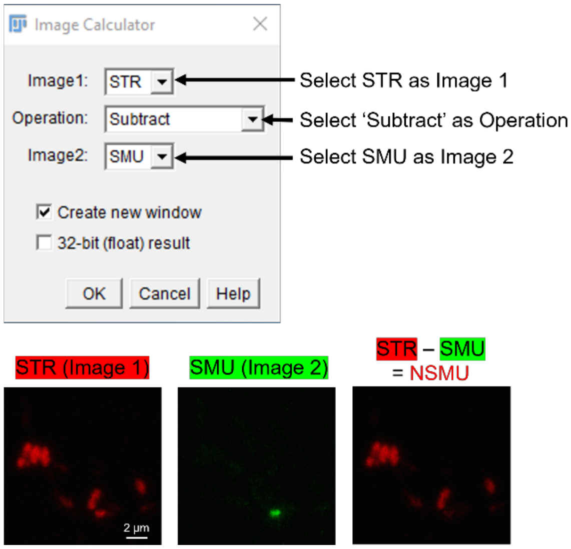

SI Methods Fig. 7. Subtraction of SMU (*S. mutans*) from STR (*Streptococcus*).

1.2.1.10. Step 5: Save the processed image stack in a separated folder (named NSMU and NSTR).

1.2.1.11. Step 6: Subject voltex rendering of the image using Amira software (Visage Imaging): *File→Open Data→Display→Voltex→Colormap (selection of color; e.g., green for S. mutans (SMU))→Apply* (tutorial is available at http://www1.udel.edu/ctcr/sites/udel.edu.ctcr/files/Amira%20Users%20Guide.pdf).

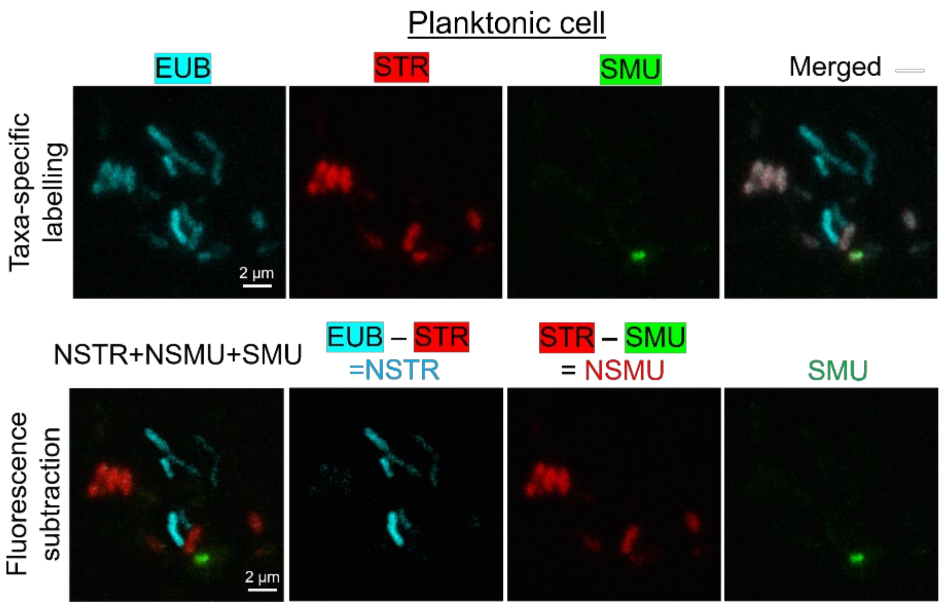

SI Methods Fig. 8. Application of taxa-specific labelling and fluorescence subtraction methods in planktonic cell mixture.

1.2.1.12. Upper panel images reveal that the taxa-specific labelling of planktonic cell mixture of *S. mutans*, *S. oralis* and *A. naeslundii*.

1.2.1.13. Bottom panel images show how fluorescence subtraction can separate non-streptococcal bacteria (i.e., *A. naeslundii*, depicted in blue), non-mutans streptococci (i.e., *S. oralis,* depicted in red), and *S. mutans* (species-specifc labelled cell, depicted in green) from planktonic cell mixture via taxa-specific labelling (SI Methods Fig. 8).

1.2.1.14. Subtraction of STR from EUB is non-streptococcal bacteria (NSTR) (*A. naeslundii* is labelled by EUB only while *Streptococcus* genus (i.e., *S. mutans and S. oralis*) is labelled by STR and EUB).

1.2.1.15. Subtraction of SMU from STR is non-mutans streptococci (NSMU) (since both *S. mutans* and *S. oralis* are labelled by STR and EUB but *S. oralis* is not labelled by SMU).

1.2.2. Mixed-species biofilm

1.2.2.1. Prepare a three-species *in vitro* biofilm. The inoculum is prepared with *S. mutans*, non-mutans streptococci (*S. oralis*), and non-streptococcal bacteria (*A. naeslundii*).

1.2.2.2. Each of the bacterial suspension is mixed to provide an inoculum with a defined microbial population of *S. mutans* (10^5 CFU ml^{-1}), *S. oralis* (10^7 CFU ml^{-1}) and *A. naeslundii* (10^6 CFU ml^{-1}).

1.2.2.3. The mixed population is inoculated in 2.8 ml of UFTYE containing 0.1% w/v) sucrose to form the initial biofilm community on the saliva-coated hydroxyapatite discs (sHA; surface area, 2.7 ± 0.2 cm^2) for 19 h. Then, the biofilms are transferred to UFTYE containing 1% sucrose to induce environmental changes to simulate a cariogenic challenge at 19 h. At 29 h, the biofilm is labelled with taxa-specific probes through FISH.

1.2.2.4. Wash the mixed-species biofilms formed on sHA gently with PBS twice.

1.2.2.5. Fix the sample with 4% paraformaldehyde (in PBS, pH 7.4) at 4°C for 4 h.

1.2.2.6. After washing with PBS, transfer the sample to 50% ethanol (in PBS, pH 7.4) and store at −20°C.

1.2.2.7. Incubate in the hybridization solution (25% formamide, 0.9 M NaCl, 0.01% SDS, 20 mM Tris-HCl, pH 7.5) containing FISH oligonucleotide probes as described in the previous section (1.1.9–1.1.10).

1.2.2.8. Wash with PBS twice and subject to confocal imaging (LSM 800 (Zeiss)) equipped with a 20× (1.0 NA) water immersion objective.

1.2.2.9. After image acquisition, fluorescence subtraction is applied as described above (from 1.2.1.4 to 1.2.1.11).

1.2.2.10. Subtraction of STR from EUB is non-streptococcal bacteria (NSTR). *A. naeslundii* is labelled by EUB only while *Streptococcus* genus (i.e., *S. mutans and S. oralis*) is labelled by STR and EUB (SI Methods Fig. 9).

1.2.2.11. Subtraction of SMU from STR is non-mutans streptococci. Both *S. mutans* and *S. oralis* are labelled by STR and EUB but *S. oralis* is not labelled by SMU.

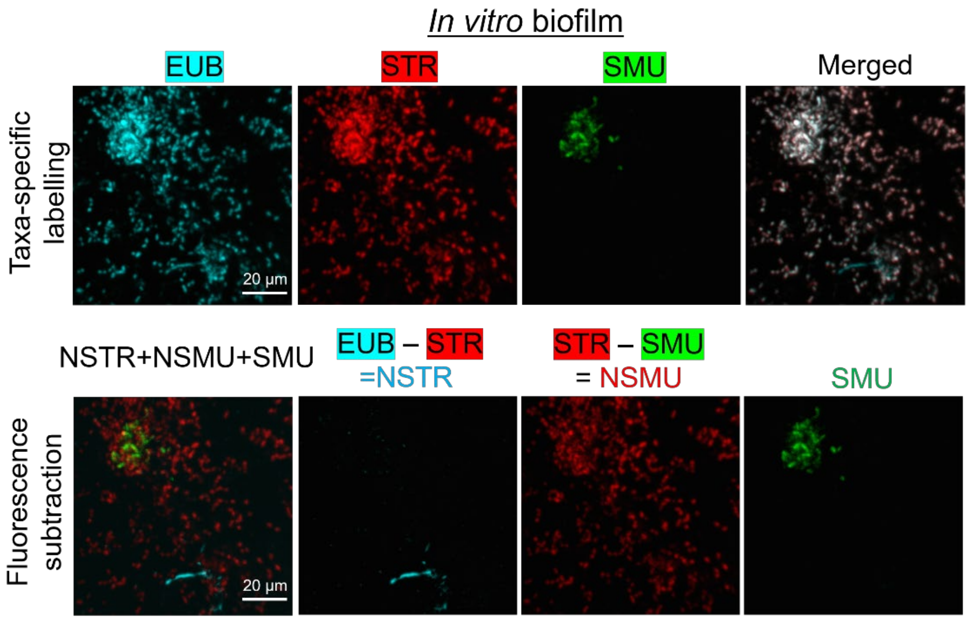

SI Methods Fig. 9. Application of taxa-specific labelling and fluorescence subtraction methods in a mixed-species biofilm.

1.2.3. ***Intact plaque biofilm on the tooth surface***

1.2.3.1. The tooth samples are fixed for intact biofilm imaging in its native state.

1.2.3.2. Apply taxa-specific labelling and multi-scale imaging (following taxonomic ranks of bacteria (domain-genus-species) and multiple length scale (overall organization (submillimeter)-architecture (micron)-structural organization (submicron)).

1.2.3.3. Acquired images are subjected to the image processing (following the procedure described above; from 1.2.1.4 to 1.2.1.11).

1.2.3.4. Subtraction of STR from EUB is non-streptococcal bacteria (NSTR) while subtraction of SMU from STR is non-mutans streptococci (NSMU) (SI Methods Fig. 10).

1.2.3.5. The fluorescence subtraction of taxonomically labelled bacteria reveals a unique bacterial cell arrangement (corona-like) (SI Methods Fig. 11).

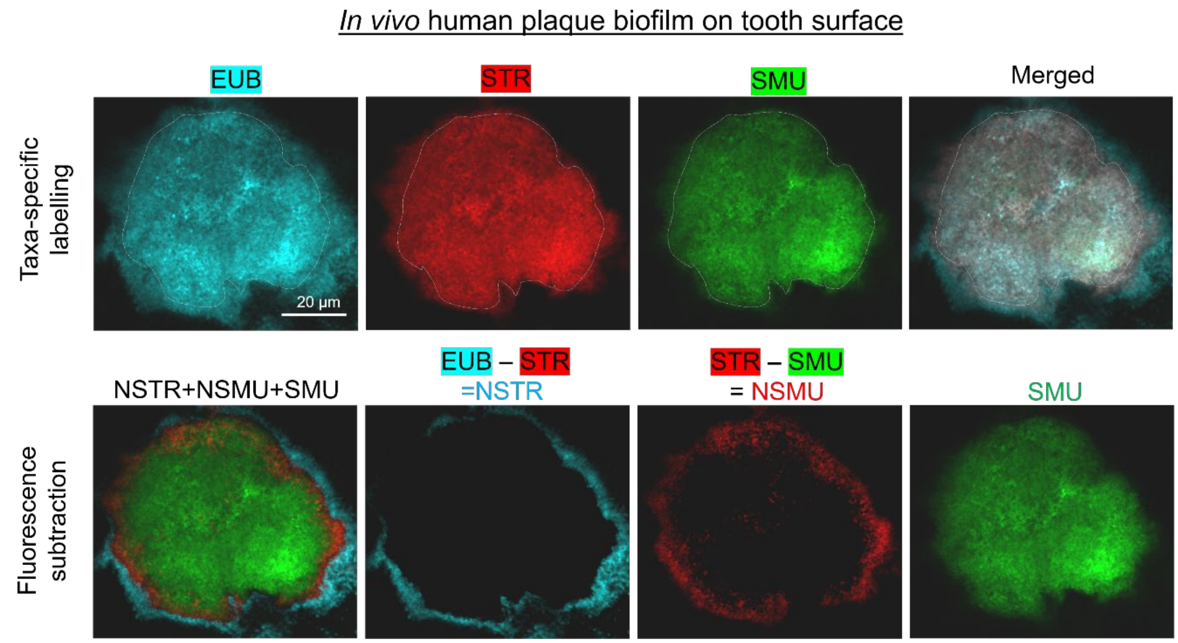

SI Methods Fig. 10. Application of taxa-specific labelling and fluorescence subtraction methods for *in vivo* human plaque biofilm on tooth surface.

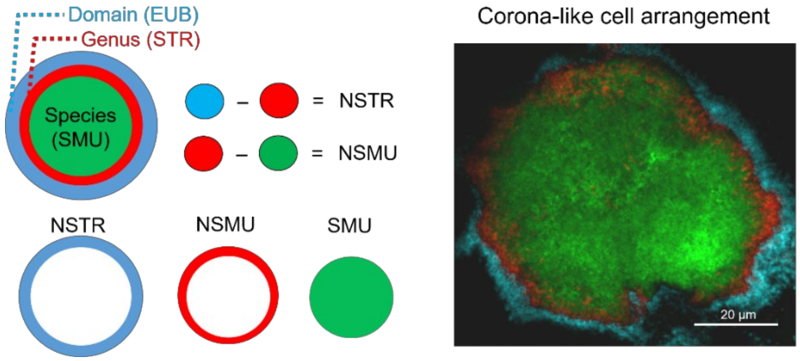

SI Methods Fig. 11. A fluorescence subtraction method for the configuration of polymicrobial community organization (corona-like cell arrangement) in intact plaque biofilm.

2. Image alignment for synchronized biofilm-surface analysis

2.1. *Biofilm-surface image acquisition: Sequential image acquisition and image realignment*

2.1.1. Prior to bacterial inoculation for biofilm growth, wide-field images of the entire enamel surface are acquired using a stereomicroscope (Axis Zoom V16, Zeiss) equipped with a 1× objective (PlanNeoFluar Z, Zeiss) under bright field and green fluorescence.

2.1.2. After biofilm formation, the wide-field images of entire biofilm formed the enamel block (4x4 mm^2) are acquired using stereomicroscope (Zeiss) for optical alignment of biofilm-surface.

2.1.3. Then, high resolution images are acquired using confocal microscopy LSM 800 (Zeiss) equipped with a 20× (1.0 NA) water immersion objective.

2.1.4. After biofilm imaging using the hybrid confocal-stereomicroscopy system, biomass is removed with an enzymatic treatment (mixture of 8.75 units of dextranase and 1.75 units of mutanase) at 37°C for 2 h followed by water-bath sonication (for 4 min).

2.1.5. The cleaned enamel surface is subjected to optical and fluorescence imaging via stereomicroscope (Zeiss).

2.1.6. Using acquired images in a sequential manner (surface imaging before biofilm culturing→biofilm imaging→surface imaging after biofilm removal), all biofilm-surface images are arranged in a grid through alignment of the optically defined edges (distinctive edges as fiduciary marks) of enamel blocks (SI Methods Fig. 12) as follows:

2.1.7. Step1: Define the distinctive edges of specimen (from biofilm-surface imaging).

2.1.8. Step 2: Arrange the images in a grid box (4.7 mm× 4.7 mm; 1 mm major gridline and 0.1 mm minor gridline).

2.1.9. Step 3: Select the area of interest through the mapping of the biofilm and the enamel surface position followed by the image alignment.

2.1.10. Step 4: Crop the images from the area of interest.

2.1.11. Step 5: Realign all the images (SI Methods Fig. 13).

2.1.12. Step 6: Evaluate the association of biofilm structure (marked with lines) on the enamel demineralization (delineated areas of eroded enamel with opaque and white chalky appearance) underneath the biofilm.

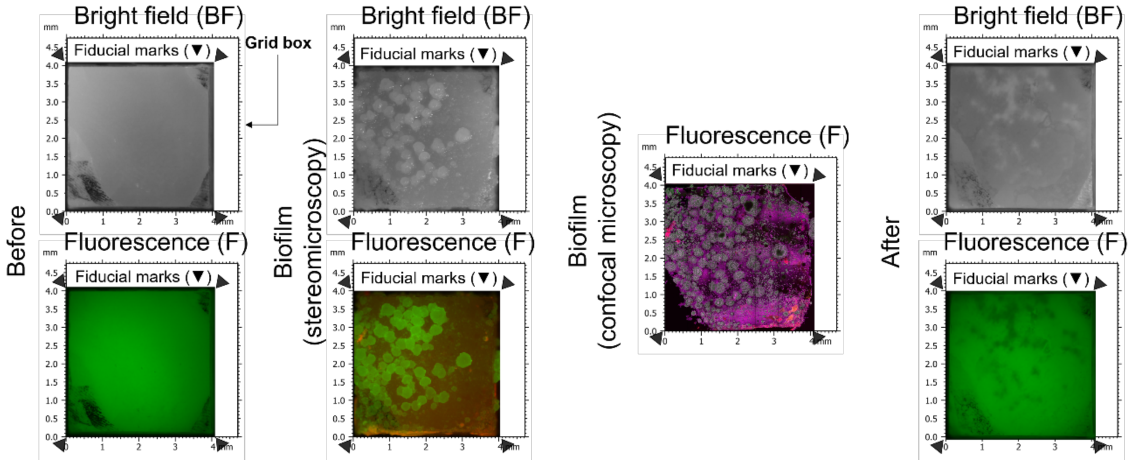

SI Methods Fig. 12. Sequential image acquisition for biofilm-surface alignment.

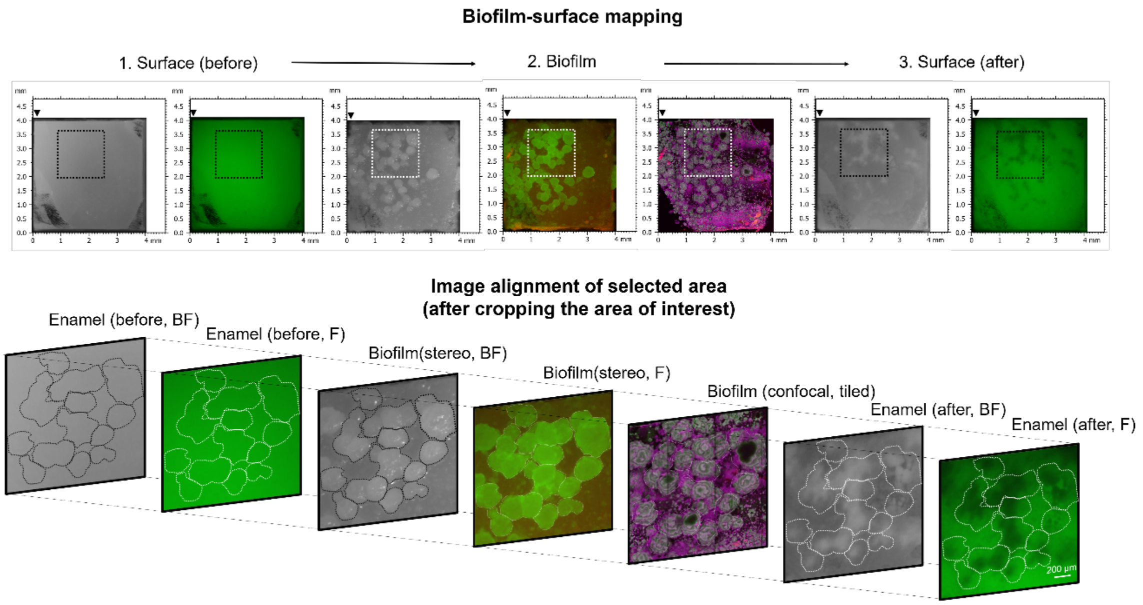

SI Methods Fig. 13. Biofilm-surface mapping for image realignment of the area of interest.

2.2. Transversal microradiography (TMR): Measurement of demineralized lesion depth

2.2.1. All acquired images (before and after biofilm removal) are realigned using the edge of enamel blocks. To match community structures (e.g., rotund) and demineralized enamel lesions underneath the biofilm, acquired images are aligned using a grid box (described in section 2.1).

2.2.2. After image realignment, lesion depth of the demineralized enamel regions is determined using TMR as described below:

2.2.3. <u>Step 1</u>: Select the area of interest (after biofilm-surface image realignment) (highlighted in green-colored box, SI Methods Fig. 14).

2.2.4. <u>Step 2</u>: Map the location (y-axis section) of the area of interest in a grid box (4.7 mm× 4.7 mm; 1 mm major gridline and 0.1 mm minor gridline).

2.2.5. For example, the entire cross-section of the enamel block encompassing both demineralized and non-demineralized areas is selected (a dotted-line box in SI Appendix Fig. 14).

2.2.6. <u>Step 3</u>: Mount specimen on a plastic rod and section with a hard tissue microtome (cut from 200–300 μm from left and right sides of the area of interest).

2.2.7. <u>Step 4</u>: A 100-μm section (encompassing the area of interest) is mounted on X-ray sensitive plates (Microchrome Technology) along with an aluminum calibration step wedge. The plates are developed according to the manufacturer's instructions.

2.2.8. <u>Step 5</u>: The section is subjected to Ni-filtered Cu-Kα radiation (X-ray; Philips Electronic Instruments) at 30 mA and 20 kV for 65 min.

2.2.9. <u>Step 6</u>: Microradiographic images are analyzed with dedicated software (TMR 2000, Inspektor) with sound enamel defined at 87% mineral volume to obtain mean lesion depth (μm). Two parameters are measured; mineral content loss (% volume mineral × μm) and lesion depth (μm).

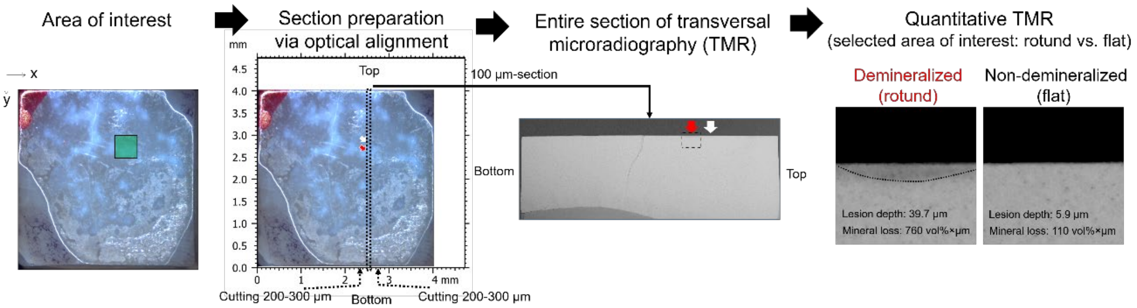

SI Methods Fig. 14. Biofilm-surface mapping for transversal microradiography.

References

1. H. Koo, J. Xiao, M. I. Klein, J. G. Jeon, Exopolysaccharides produced by Streptococcus mutans glucosyltransferases modulate the establishment of microcolonies within multispecies biofilms. *J. Bacteriol.* **192**, 3024–32 (2010).
2. J. Xiao, *et al.*, The Exopolysaccharide Matrix Modulates the Interaction between 3D Architecture and Virulence of a Mixed-Species Oral Biofilm. *PLoS Pathog.* **8**, e1002623 (2012).
3. G. Hwang, *et al.*, Simultaneous spatiotemporal mapping of in situ pH and bacterial activity within an intact 3D microcolony structure. *Sci. Rep.* **6**, 32841 (2016).
